# Grafting enhances drought tolerance by regulating and mobilizing proteome, transcriptome and molecular physiology in okra genotypes

**DOI:** 10.3389/fpls.2023.1178935

**Published:** 2023-05-12

**Authors:** Kaukab Razi, Sowbiya Muneer

**Affiliations:** ^1^ Horticulture and Molecular Physiology Lab, Department of Horticulture and Food Science, School of Agricultural Innovations and Advanced Learning, Vellore Institute of Technology, Tamil Nadu, Vellore, India; ^2^ School of Biosciences and Technology, Vellore Institute of Technology, Vellore, Tamil Nadu, India

**Keywords:** drought resistance, grafting, okra genotypes, physio-morphogenesis, proteomics, transcriptome

## Abstract

Drought stress poses a serious concern to the growth, development, and quality of the okra crop due to factors including decreased yield, inadequate development of dietary fibre, increased mite infestation, and decreased seed viability. Grafting is one of the strategies that have been developed to increase the drought stress tolerance of crops. We conducted proteomics, transcriptomics and integrated it with molecular physiology to assess the response of sensitive okra genotypes; NS7772 (G1), Green gold (G2) and OH3312 (G3) (scion) grafted to NS7774 (rootstock). In our studies we observed that sensitive okra genotypes grafted to tolerant genotypes mitigated the deleterious effects of drought stress through an increase in physiochemical parameters, and lowered reactive oxygen species. A comparative proteomic analysis showed a stress responsive proteins related to Photosynthesis, energy and metabolism, defence response, protein and nucleic acid biosynthesis. A proteomic investigation demonstrated that scions grafted onto okra rootstocks increased more photosynthesis-related proteins during drought stress, indicating an increase in photosynthetic activity when plants were subjected to drought stress. Furthermore, transcriptome of *RD2*, *PP2C*, *HAT22*, *WRKY* and *DREB* increased significantly, specifically for grafted NS7772 genotype. Furthermore, our study also indicated that grafting improved the yield components such as number of pods and seeds per plant, maximum fruit diameter, and maximum plant height in all genotypes which directly contributed towards their high resistance towards drought stress.

## Introduction

Drought has a significant impact on food security particularly in rural regions with large populations of small-scale farmers who are totally dependent on rain-fed agriculture for their food and livelihoods (Jain et al., 2015). The average summer temperature in the southern area of India exceeds 40 degrees Celsius. High temperature increases leaf wilting, limits shoot and root growth, and reduces the formation of dry matter (Qaderi et al., 2012). Drought stress has become a growing issue as a significant environmental restriction due to its negative effects on crop development and productivity. When exposed to extreme temperatures, drought-tolerant plant genotypes exhibit greater photosynthetic efficiency than drought-sensitive ones (Mishra et al., 2017). Photosynthesis is a temperature-sensitive physiological process (Mishra et al., 2017). Therefore, drought stress always disrupts the expression levels of plant proteins, particularly photosynthesis-related proteins.

To resist drought stress and adapt to survival environment, plants produce a range of changes at the physiological, cellular, and molecular levels to regulate osmotic and ion homeostasis which helps to maintain their development and growth, such as changes in morphology (Rahman et al., 2016), antioxidant enzymes (Mittler et al., 2011), and osmoregulation (Fu et al., 2016). Plants can improve their drought tolerance by increasing the activity of antioxidant enzymes and the expression of their related genes under drought stress (Sandhu et al., 2017). Antioxidant enzymes like catalase, ascorbate peroxidase (APX), superoxide dismutase (SOD), are crucial in scavenging those reactive oxygen species produced in the plants (Gill and Tuteja, 2010). Under drought stress, different plant species accumulate sugars (such as glucose, fructose, and sucrose), sugar alcohols (such as mannitol), and amino acids (such as proline), which serve not only as osmolytes but also as antioxidants, assisting in ROS detoxification, membrane protection, and enzyme/protein stabilisation, ultimately enhancing plant resistance to abiotic stresses, (Seki et al., 2007). Among these metabolites, changes in proteins are an important signalling process in plant growth and development. The study of global protein expression is made easier by proteomics analysis, which also offers a wealth of knowledge about the function of individual proteins in many biological processes. Proteomics is a reliable and reproducible high-throughput approach in understanding biological processes (Ahmad et al., 2012). Proteomic studies can analyse and document hundreds of protein expressions at a single time of organisms at any specific time. There have been many studies on proteomic changes in response to abiotic stress in plants such as Arabidopsis (Jiang et al., 2007), wheat, (Guo et al., 2012), maize (Zörb et al., 2010), and cucumber (Du et al., 2010). However, no proteomic information from grafted okra has been published till yet. Our thorough research offers useful data which helps to investigate the functions of potential proteins in reducing the harm brought on by drought stress in okra.

A member of the Malvaceae family and a highly nutritious underused crop, okra (*Abelmoschus esculentus* [L.] Moench) is grown extensively in India (Shah and Nahakpam, 2012). While mature and dry seeds are a rich source of edible oils and fatty acids (abundant in Protein (22.14%), amino acids (lysine and tryptophan), fibre, vitamins (A, C, and K), and minerals (calcium, potassium, salt, and magnesium) are all abundant in the seeds, tender and immature pods are consumed as a vegetable (Thirupathi Reddy et al., 2013). Consumption of okra has various positive effects on human health, including lowering blood sugar levels, lowering cholesterol, and alleviating constipation (Gemede et al., 2015). Furthermore, the production of okra is severely hampered by biotic (such as the yellow mosaic virus) and abiotic stress (such as drought and heat). About 30 to 100% yield loss have been reported in okra particularly due to drought stress (Mbagwu and Adesipe, 1987). Considering the importance of okra, the grafted okra genotypes were also tested for yield study in randomized design (pot study) and were carried out on the basis of the number of pods and seeds, plant height, stem girth etc. Therefore, keeping the above points in mind, a research work has been formulated with an objective to evaluate yield performance of grafted okra genotypes to support the farmers in okra cultivation. Prior to now, very few genotypes of okra that could withstand drought stress have been investigated (Shi et al., 2020). Additionally, there are fewer breeding initiatives to create genotypes of drought-tolerant okra for production in arid and semi-arid regions around the world. Based on their genomic data, previous research has discovered genes associated with drought tolerance in rice, wheat, and a few other plant models. However, very limited information is available regarding the drought-resistant genes in grafted okra genotypes till date. In order to determine the gene expression patterns of grafted okra and to pinpoint drought-tolerant genes, we analysed the transcriptome of grafted okra under drought stress conditions in this work which would help deepen our comprehension knowledge of how okra adapts to drought.

However, plants' natural drought stress responses aren't always enough to guarantee plant survival under drought stress situations. Numerous methods have been used to overcome this, and most lately, the use of grafting as a sustainable method has come to light. Grafting, an ancient technique, is one of the most important methods for the vegetative propagation of horticultural crops, such as vegetables, fruits, and flowers (Melnyk and Meyerowitz, 2015). Grafting can enhance a plant's tolerance to environmental stress, disease and worm resistance, and fruit quality and yield (Kubota et al., 2008). Vegetable grafting has benefited numerous plant species, including eggplants, tomatoes, peppers, watermelons, cucumbers, and melon. Grafting-mediated drought tolerance is likely the result of increased photosynthetic capacity, carbon assimilation rate, and antioxidant enzyme capacity (Javid et al., 2011). However, it remains unclear how drought tolerant rootstocks regulate okra resistance under drought stress and how grafted and non-grafted okra differ in their response to drought stress.

In the present study, a proteomics-based methodology (SDS PAGE accompanied by LC MS/MS) was used to explore the effect of drought stress on grafted okra genotypes and to discover differentially accumulated proteins regulated by rootstock and drought stress. We found that grafting drought sensitive okra scion onto tolerant okra rootstock boosted their drought tolerance, which was accompanied by increased chlorophyll content, leaf area, and photosynthesis. In response to rootstock-grafting under drought stress, differently accumulated proteins were discovered and categorised according to their biological functions including Photosynthesis (25.1%), metabolic process (53.2%), defence response (18%), Metal binding (24.1%), oxidoreductase activity (11.2%) and ATP Binding (17.2%). In addition, RT-PCR was used to examine the transcript level of genes associated to drought stress responses (*RD2, HAT22, PP2C, DREB1A, DREB1C and WRKY33*) which provided further validation to our results. Our results provide evidence, from both physiological and transcriptomic perspectives that grafted okra enhance drought tolerance through changes in reactive oxygen species (ROS), soluble sugar, and proline pathways. This current research helped us to understand the mechanisms by which grafting improves the drought tolerance in okra, making it possible to cultivate them on a large scale in drought affected areas.

## Materials and methods

### Plant materials and growth conditions

The experiment was conducted in polyhouse of School of Agricultural Innovations and Advanced Learning (VAIAL), Vellore Institute of Technology, Vellore, India. The following were the polyhouse's growing conditions: Temperature regime of 28°C and 25°C Day and night respectively, and relative humidity of roughly 90 ± 5%. okra (*Abelmoschus esculentus* L.) seeds of four different genotypes that are NS7774, NS7772, green gold and OH3312 were collected from local vendors in Vellore (company name: Namdhari and Syngenta). The seeds were sown in protrays; each portrays having 50 wells and each well occupying one okra seed for each genotype (**Supplementary Figures 1**, **2**). The protrays were filled with red soil and vermicompost in the ratio 1:1 and the soil pH were 6.86.

### Plant grafting and drought stress treatment

The experiment was arranged in a completely randomized design (CRD) such that each treatment had five replicates. Three combinations of grafted plants were prepared: NS7772 grafted onto NS7774 (G1), green gold grafted onto NS7774 (G2) and lastly OH3312 grafted onto NS7774 (G3) as NS7774 is the drought tolerant rootstock as previously studied (Razi et al., 2021).The seeds were sown at the appropriate planned time, so that once the vegetative stage arrived, 25-30 days after date of sowing and if the rootstock was vigorous enough then grafting was carried out in the early morning timings using cleft grafting method (**Supplementary Figure 3**). All the homograft was covered with transparent plastic to maintain the humidity at 90%–95% and temperature at ∼25–28°C to accelerate the healing process and avoid water loss. Two week later, when the grafted plants had survived and the graft junctions healed, healthy and uniform plants with similar size were chosen and transferred to grow bags. Each bag contained two plants and similar content of soil as mentioned above was added. Overall, twenty-eight days after grafting, the grafted plants (G1, G2 and G3) were divided into two treatment sets having one as control condition and the other as drought stress condition groups. The water content in drought stress treated grafted plants was gradually decreased by withholding irrigation for 10 consecutive days. Leaf samples were collected after 10 days of treatment and stored at −80°C for further experimentation.

### Morphological observation of plant roots and shoot

The root and shoot of all the grafted plants (G1, G2 and G3) were cleaned with an agricultural compression sprayer to observe the morphology by taking photographs. The root length and shoot length were measured after 5 and 10 Days of drought stress. The roots and shoots of the plant were separated to determine the fresh weight and then dried in hot air oven at 65°C for 24 h to determine the dry weight.

### Leaf relative water content

Leaf relative water content (RWC) was determined according to the standard method proposed by Barrs and Weatherley, 1962 and calculated as RWC=(FW−DW)/(TW−DW), where FW is fresh leaf weight, DW is dry weight and TW is turgid weight.

### Gas exchange parameters, Fv/Fm, pigment content

The photosynthetic rate, stomatal conductance, transpiration rate and Fv/Fm of individual leaf blades were measured between 9:00 and 12:00 am using a portable SPAD meter (Konika Minolta, Tokyo, Japan). PAM 2000 chlorophyll fluorescence meter (Heinz Walz GmbH, Zarges 40860, Weilheim, Germany) was used to measure chlorophyll fluorescence (Fv/Fm). The maximum PS II quantum yield (Fv/Fm) was calculated as Fv/Fm = (Fm − F0)/Fm. For all these experiments, the topmost fully expanded leaves were used. The same samples were used for pigment content (Total chlorophyll content and carotenoid) determination as described by Hiscox and Israelstam (1979) and were calculated using the Arnon methods.

### Water transport activity

Fresh plant samples were uprooted and washed properly. They were fixed in 0.1% food-coloring agent for 5–6 h and then rinsed with water. The stem of every genotype was cut into transverse sections with the help of a sharp surgical razor-blade and was observed under a dark field and phase contrast microscope (model: MT4300L, MEIJI TECHNO CO., LTD., Kyoto, Japan).

### Structure of stomata and stomatal index

Fresh and healthy leaf chosen and folded to gently pull the peel apart to separate a peeled section from the lower surface of the leaf using the forceps. For a few minutes the peel was allowed to remain in a watch glass containing water. The sample was further stained by adding few drops of safranin for about 2-3 minutes, later a drop of glycerine was added using a needle. The structure of stomata was visualized under high power magnification by using compound microscope. The same samples were used for stomatal index determination as described previously (Razi et al., 2021).

### Determination of hydrogen peroxide, MDA content and relative electrolyte leakage

The topmost leaf samples were acquired at 5 and 10 DAS. The harvested samples were promptly frozen in liquid nitrogen then stored in a refrigerator at −80°C until used for the following relevant experiments.

With a few minor modifications, H_2_O_2_ content was determined in accordance with Velikova et al., 2000. Fresh leaves measuring 0.25 g were homogenised in 2 ml of 0.1% (w/v) trichloroacetic acid (TCA) before being centrifuged at 4°C at 10,000 rpm for 8 minutes. Approximately 0.6 ml of 0.1% (w/v) TCA was added to the supernatant (0.4 ml), and the samples were incubated for one hour in the dark at room temperature and absorbance was measured at 390 nm. The H_2_O_2_ standard curve was utilised to determine its concentration. Malondialdehyde content (MDA) content was performed as described previously (Razi et al., 2021). The protocol described by Lutts et al., 1996 served as the basis to calculate the percentage of electrolyte leakage. To remove surface impurities, leaf samples were cleaned thrice with distilled water before being put into individual stoppered vials with 10 ml of distilled water. The samples were placed on shaker for 24 hours at room temperature (25°C) at a speed of 100 rpm. Using a conductivity metre (EC tester 11+, Fieldscout, 2265FS), the electrical conductivity of the bathing solution (EC1) was assessed following incubation. Following a 15-minute incubation period in a hot water bath (95°C), samples were read for electrical conductivity (EC2) after the bathing solution had cooled to room temperature. ELP was calculated as EC1/EC2 and expressed as %.

### *In situ* localization of oxidative stress markers (H_2_O_2_ and O_2_^−^)

According to Muneer et al., 2016 the histochemical staining of hydrogen peroxide (H_2_O_2_) and superoxide radicle ( ${\text{O}}_{2}^{\;-}$ ) was carried out. For H_2_O_2_ localization, fresh leaf samples were soaked in a 0.1% solution of 3, 3′-diaminobenzidine (DAB) in Tris-HCl buffer (pH 6.5). Five minutes of vacuum infiltration was followed by a 12-hour incubation period in the dark. The leaves were then bleached in boiling ethanol (95%) and photographed using a digital camera to detect the brown spots. Fresh leaf samples were immersed in a 0.1% solution of nitro blue tetrazolium chloride (NBT) in a K-phosphate buffer (pH 6.4) containing 10 mM sodium azide in order to determine the location of oxygen. The subsequent processes were carried out in the same way as the H_2_O_2_ localization. The development of blue formazan precipitate was seen and photographed using a digital camera.

### Determination of proline content, total soluble protein, and total soluble sugars

Proline content was determined according to Bates et al., 1973 with minor modifications. To quantify total soluble protein content, 0.5 g of leaf sample was homogenised in 1 ml of 200 mM Phosphate buffer (pH 7) and centrifuged at 8000 rpm for 10 minutes. 0.5 ml of supernatant was added to 10% TCA and centrifuged at 3300 rpm for 30 min. Supernatant was eliminated, pellet rinsed with water, and 0.1 N NaOH was added. Further, 0.2 ml of supernatant was combined with 5 ml of Bradford reagent and incubated for 5 min. The total soluble sugar was determined according to the protocol described by Xu et al., 2021. Approximately 0.5 g of leaf samples were homogenised in 5 ml of 80% ethanol, and centrifuged at 6000 rpm for 15 min to determine the total amount of soluble sugar present. Around 12.5 ml of 80% ethanol and 1 ml of a 0.2% anthrone solution were added to the supernatant. For 10 minutes, the reaction was heated in a water bath to 100°C. At 620 nm, the absorbance was measured.

### Estimation of antioxidants enzyme activity and their relative staining

The leaf samples collected on the 5^th^ and 10^th^ day of the drought stress were used to determine superoxide dismutase (SOD), Ascorbate peroxidase activity (APX) activity and catalase (CAT) activity as described previously by Razi et al., 2021. The Superoxide dismutase (SOD) activity was determined by nitro blue tetrazolium (NBT) inhibition method as described by Giannopolitis and Ries, 1977, with slight modifications. Catalase (CAT) activity was carried according to Manivannan et al., 2016, with minor changes. Total APX activity in plant tissues was estimated by the method of Nakano and Asada, 1981, with slight modifications. For native staining, all the antioxidant enzymes (30 µg of protein) were electrophoresed at 4°C for 4 hours at 80 volts in Tris-Glycine (pH 8.3) running buffer. The APX and CAT isozymes were separated using 10% resolving gel, while the SOD isozymes were separated using 15% resolving gel (Shah and Nahakpam, 2012). Gels were soaked in 50 mM potassium phosphate buffer (PPB) (pH 7) with 2 mM ascorbate for 30 minutes prior to APX staining. Gels were then incubated in 50 mM PPB (pH 7) for a further 20 minutes with 4 mM ascorbate and 2 mM H_2_O_2_, rinsed in 50 mM PPB (pH 7) for 1 minute, and then placed in 50 mM PPB (pH 7.8) with 28 mM TEMED and 2.45 mM NBT while being gently stirred. Bands were then seen under white light. Gels were submerged in 3.27 mM H_2_O_2_ for 25 minutes to perform CAT staining, and then they were quickly rinsed with distilled water for one minute. The gel was then further dyed for 10-15 minutes with 1% ferric chloride and 1% potassium ferricyanide. Following staining, the gel was cleaned and exposed to white light until bands formed. Gels were pre-equilibrated in 50 mM PPB (pH 7.8) with 1 mM EDTA and 5 mM H_2_O_2_ for SOD labelling. The gel was then incubated for 1 hour in the dark with 50 mM PPB (pH 7.8), 0.24 mM NBT, 33.2 M riboflavin, and 0.2% TEMED. White light was shown onto gels until bands started to form.

### Native PAGE profiling of isozymes of peroxidases’ enzyme(s)

According to the protocol described by Lee et al., 2007, Fresh samples (0.5 g) were homogenized in 1.5 ml of 100 mM K-PO_4_ buffer solution (pH 7.0) containing 2 mM phenylmethylsulphonyl fluoride, and centrifuged at 14 000 g for 20 minutes at 4°C to extract the enzymes. In order to retain the enzymatic activities after extraction, all the protein samples were prepared in a modified Laemmli buffer without SDS and dithiothreitol and 5% stacking gel and 10% or 12.5% resolving gel was prepared and used for protein separation. For active staining of GPOX after PAGE, gels were incubated with 0.46% (v/v) guaiacol solution and 13 mM H_2_O_2_ in 50 mM Tris-Cl buffer at room temperature until red bands appeared. For SPOX staining, syringaldazine was dissolved in 30 ml of methanol (3.6 mg ml^-1^) and then mixed with dioxin (1:2), 2.5 mM H_2_O_2_ and 30 ml of 0.1M Na-K phosphate buffer (pH 6.0).

### First dimensional gel electrophoresis (SDS-PAGE)

The plant tissue (1g) was extracted using freshly prepared protein extraction buffer as previously described by Muneer et al., 2016. The extraction buffer constituted (pH 7.5) 40 mM (*w/v*) Tris-HCl (pH 7.5), 2 mM (*w/v*) EDTA, 0.07% (*w/v*) β-mercaptoethanol, 2% (*w/v*) PVP and 1% (*v/v*) Triton X-100. The extract was centrifuged at 13,000 rpm for 10 min at 4°C. The supernatant was mixed with protein-dye and 20 µg proteins were loaded on 12.5% polyacrylamide gel on PROTEAN II (Bio-Rad, Hercules, CA, USA). The protein concentration was determined by the Bradford method using BSA (bovine serum albumin) as a standard curve. Following electrophoresis, the gel was stained with commercially available Coomassie brilliant blue stain (CBBS) according to manufacturer’s instruction (Bio-Rad).

### In-gel digestion of protein bands and mass spectrometer analysis

The protein bands were manually excised using a clean razor blade and distilled water and further the in-gel digestion was carried out according to Muneer et al., 2016. Protein bands from 1DE gels were manually excised with a clean razor blade and rinsed three times in distilled water. The bands were further incubated for 30 minutes at room temperature in a de-staining solution made of 30 mM potassium ferricyanide and 100 mM sodium thiosulphite pentahydrate (1:1). After draining the de-staining solution, the gel pieces were treated with 100 mL of 50 mM NH_4_CO_3_ and then dehydrated for 5 minutes in 30 mL of acetonitrile. The gel fragments were dehydrated before being subjected to 100 L of a reduction solution (10 mM dithiothreitol in 50 mM NH_4_CO_3_) and being incubated at 56°C for 45 minutes. After draining the reduction solution, 100 L of the alkylation solution was incubated at 25°C for 30 minutes (100 mM iodoacetamide in 50 mM NH_4_CO_3_). The gel fragments were then dehydrated for 10 minutes in 30 mL of acetonitrile after being rinsed in 30 mL of 50 mM NH_4_CO_3_. The gel fragments were vacuum centrifuged dried, and then rehydrated for 30 minutes at 37°C in 5–10 L of 25 mM NH_4_CO_3_ containing 5 ng/L trypsin (Promega, Madison, WI, USA). After 16 hours of digestion at 37 degrees Celsius, the excess trypsin solution was replaced with 5 to 10 L of 25 mM NH_4_CO_3_, and the digested peptides were collected, dried under vacuum, and combined with 3 L of 50% acetonitrile and 0.1% trifluoroacetic acid.

Using a mass spectrometer (Bruker Ultraflex III MALDI-TOF/TOF Mass Spectrometer, Bruker, Massachusetts, USA), materials that had been trypsin digested were examined. 2 l of a 1:1 (v/v) mixture of tryptic digest and matrix solution (10 mg/ml R-cyano-4-hydroxycinnamic acid (CHCA) in 50% ACN/0.1% TFA) were spotted onto the designated plate. 1000 laser shots (summed/averaged) were used to create each mass spectrum, which was then used to gather mass spectra in reflector positive ion mode with an accelerating voltage of 21 kV spanning the mass range of 700-3000 Da. For external calibration, a mixture of ACTH and angiotensin standards from Sigma-Aldrich in St. Louis, Missouri, the United States, were employed. As the peptide mass fingerprint (PMF) per location, monoisotopic peaks with S/N > 5 were chosen. By averaging 2000 laser shots (summed/averaged) per fragmentation spectrum, parent ion spectra (from TOF/TOF fragmentation) were obtained where necessary throughout a range of 40-3000 Da.

### RT-PCR analysis

RNA isolation was performed using an RNA isolation Kit from the leaves of grafted okra genotypes as per the manufacturer’s instructions (Hi-Media). The Real Time PCR was performed in Applied Biosystems using SYBR Green 24 Chemistry (Sensifast HiRoxkit Bioline, USA for 5 min at 95°C, followed by 35 cycles consisting of 20 s at 25 95°C, 30 s at 57 °C and 30 s at 72 °C, then 10 min at 72 °C. Three different RNA preparations from independently grown plants were utilized for the RT-PCR reactions, along with three duplicates for the qPCR. The results were analysed using qBase plus 28 software 13. The gene specific primers used in this study are enlisted in **Table T1** (**Supplementary Table T1**).

### Yield study

This research was conducted from August 2022 to October 2022 in polyhouse of School of Agricultural Innovations and Advanced Learning (VAIAL), Vellore Institute of Technology, Vellore, India. The materials used in this research were the successfully grafted okra plant genotypes G1, G2 and G3 that were successfully transplanted to grow bags for yield study (**Supplementary Figures 4**, **5**). Experimental material comprised 3 grafted okra genotypes (G1, G2 and G3). All grafted genotypes were evaluated in a randomized block design with five replications. Observations that were recorded on five grafted okra plants for each genotype for every seven days included fruit diameter (mm), stem diameter (mm), plant height (cm), number of pods per plant, days required for first flowering, days required for 50% flowering, fruit length (cm) and total yield (g).

### Statistical analysis

For physiological parameters complete randomized design was utilized with three replicates. The Tukey’s studentized range test was employed to compare the means of separate replicates. Unless stated otherwise, the conclusions are predicated on differences between the means, with a significance level set at *P* < 0.05. For functional classification of proteins direct gene ontology consortium (http://www.geneontology.org/) was performed to get percent variation of identified proteins.

## Results

### Morphological and physiological response of grafted okra genotypes to drought stress

**Figure 1:**
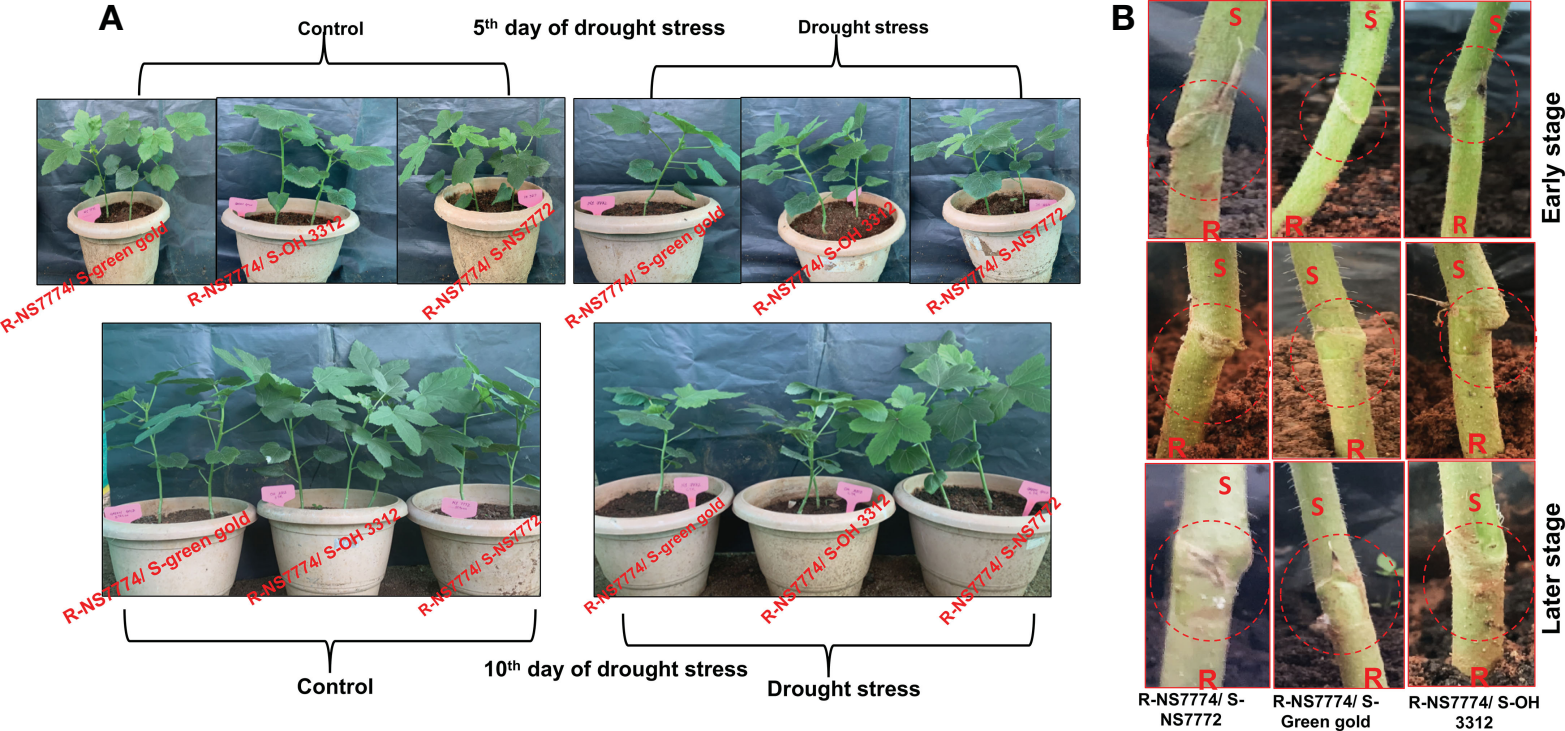
Morphological representation of **(A)** grafted okra genotypes R-NS7774/S-NS7772, R-NS77774/S-Green Gold and R-NS7774/S-OH3312. Photographs represent the early germination period, early stage and vegetative stage induced by drought stress along with control plants. A total of 5 days represent an early stage, and 10 days represent the late stages of the drought period **(B)** junction of Grafted okra genotypes R-NS7774/S-NS7772, R-NS77774/S-Green Gold and R-NS7774/S-OH3312. Photographs represent the graft junction during early stage and the later vegetative stage.

**Figure 2:**
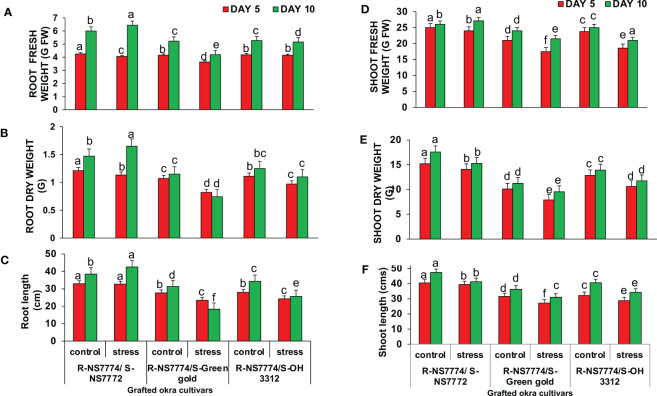
Changes in **(A)** root fresh weight, **(B)** root dry weight, **(C)** root length **(D)** shoot fresh weight **(E)** Shoot dry weight and **(F)** shoot length, as affected by drought stress at 5 and 10 days of interval in grafted okra genotypes R-NS7774/S-NS7772, R-NS77774/S-Green Gold and R-NS7774/S-OH3312 as along with their controls. Vertical bars indicate mean ± SE for n = 5. Means denoted by a different letter are significantly different at p ≤ 0.05 according to the Tukey’s studentized range test.

**Figure 3:**
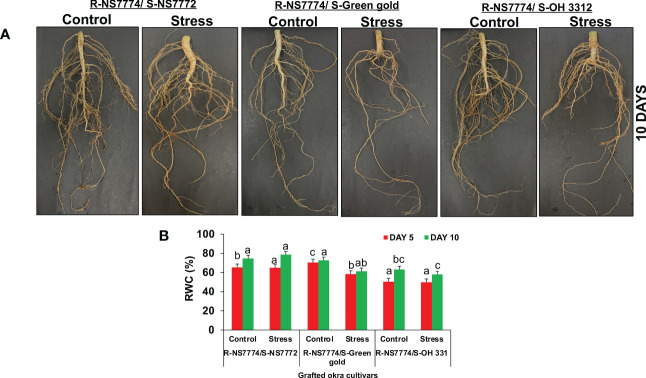
Morphological representation of **(A)** the root structure of Grafted okra genotypes R-NS7774/S-NS7772, R-NS77774/S-Green Gold and R-NS7774/S-OH3312. Photographs represent the root structure during the later vegetative stage. 10 days represent the later stages of the drought period. Changes in **(B)** relative water content as affected by drought stress at 5 and 10 days of interval in grafted okra genotypes along with respective controls. Vertical bars indicate mean ± SE for n = 5. Means denoted by the different letter are significantly different at p ≤ 0.05 according to the Tukey’s studentized range test.

To investigate whether okra genotype NS7774 as a rootstock is able to induce drought tolerance in drought sensitive scions NS7772, green gold and OH3312, we made three types of combinations of grafted plants: NS7774 as rootstock and NS7772 as scion (G1), NS7774 as rootstock and green gold as scion (G2), NS7774 as rootstock and OH 3312 as scion (G3). Then, progressive drought stress was applied for 10 days. Comparison of all homograft showed that the leaves of all the grafted okra genotypes (NS7774-rooted) stayed more hydrated and thrived better even under drought stress (**Figure 1A**). **Figure 1B** represents morphological appearance of the successfully formed graft junctions across all the grafted okra genotypes which display high percentage of graft compatibility. The highest percentage of graft success (84%) was achieved from cleft grafted okra genotype G1 (R-NS7774/S-NS7772) followed by grafted okra genotype G3 (R-NS7774/S-OH3312) and G2 (R-NS7774/S-Green gold). Next, we measured biomass which included all the growth factors like root fresh and dry weight, shoot fresh and dry weight, root and shoot length under drought stress (**Figure 2**). It is known for a fact that drought stress impairs plant growth and development in plants. Hence in our study we observed that for 5 and 10 days of drought stress, the plant root and shoot length reduced slightly for all the grafted genotypes G1, G2 and G3 (**Figures 2C, F**) except for G1 whose root length was surprisingly enhanced under drought stress. However, it could be well observed that there was maintenance in root length and shoot length of G1 under drought stress when compared to control and other grafted genotypes. A similar trend was observed for fresh and dry biomass of grafted okra genotypes (**Figures 2A, B, D, E**) where the detrimental effect of drought stress was very minimal, exclusively for homograft G1, where we observed increase in the fresh and dry biomass under 10 days of drought stress as evident in our **Figure 1A** with significantly lesser damage to leaves. Grafted okra genotype G1 showed healthier phenotype in terms of its root structure with lower root damage as compared to other genotypes (G2 and G3) under drought stress (**Figure 3A**). Relative water content (RWC) of the leaves was examined at 5 and 10 DAS for all the okra homograft (G1, G2 and G3). The results showed that the water content of the homograft G2 and G3 reduced for both 5 and 10 DAS but the percentage of decrease was comparatively lower with regard to non-grafted okra plants as previously studied. It is worth noticing that out of all, homograft G1 thrived better on the basis of highest leaf water content for both 5 and 10 days of drought stress by 5.4% and 5.3% respectively (**Figure 3B**) which could be possible due to developed root system and excellent adapting approach under drought stress.

### Grafting increased the photosynthetic pigments and water transport activity during drought stress

**Figure 4:**
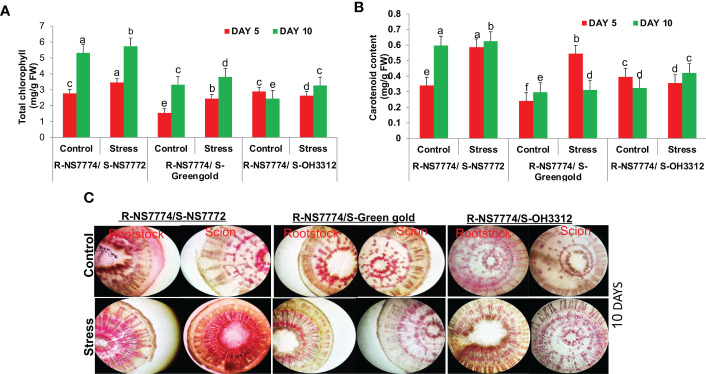
Changes in the photosynthetic pigments: **(A)** total chlorophyll content and **(B)** carotenoid, as affected by drought stress at 5 and 10 days of interval in grafted okra genotypes R-NS7774/S-NS7772, R-NS77774/S-Green Gold and R-NS7774/S-OH3312 along with respective controls. **(C)** Vascular transport, as affected by drought stress at 10 days of interval in grafted okra genotypes, along with respective controls. Red food-colouring dyes were used for the absorption process, indicating activity of vascular tissue. EP indicates epidermis; CR indicates cortex; X indicates xylem; P indicates phloem. Vertical bars indicate mean ± SE for n = 5. Means denoted by the different letter are significantly different at p ≤ 0.05 according to the Tukey’s studentized range test.

On day 10 of drought treatment, the chlorophyll content of homograft G1, G2 and G3 increased (**Figure 4A**). This showed that the use of grafting with drought tolerant rootstock NS7774 had a greater impact on improving pigment content for 10 days of drought stress than 5 days of stress which could be due to inactivation of enzymes like chlorophyllase (inhibiting enzyme) which are responsible for degradation of pigments under drought stress. A similar pattern was observed with increase in carotenoid content during drought stress upon grafting during day 5 and day 10 of treatments (**Figure 4B**). For knowing the water status of vascular bundles (xylem and phloem), water transport activity was checked using food colouring dye (**Figure 4C**). A good passage of red colour dye was observed for all the grafted okra genotypes under drought stress as compared to control, although comparatively the presence of dye was much more superior for G1. The presence of dye is imperative of appropriate and effective connections to vessels and the functional transport of water, nutrients and other important minerals between the rootstock and scion.

### Grafting improved the gaseous exchange parameters and PSII quantum yield during drought stress

**Figure 5:**
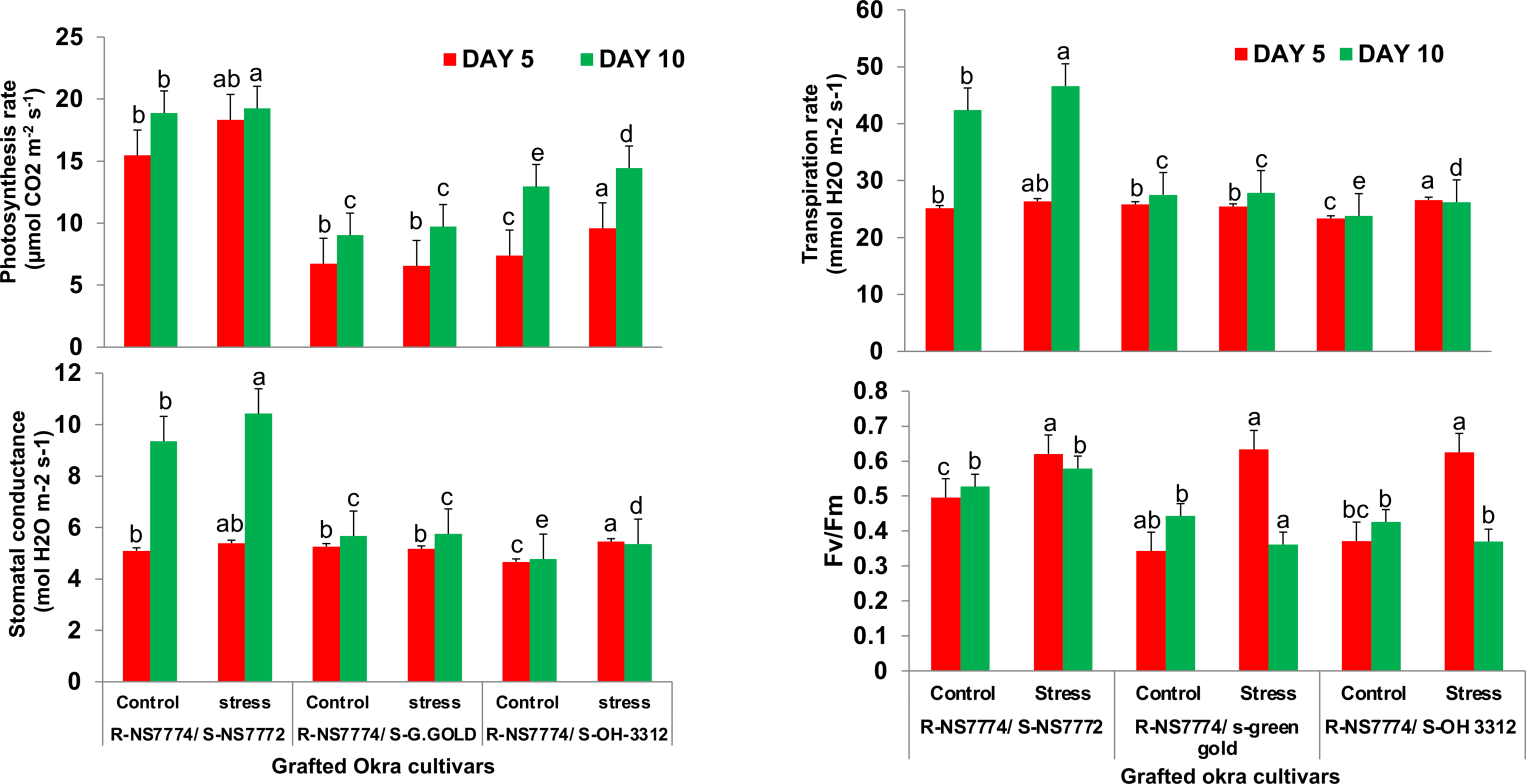
Changes in photosynthetic parameters: net photosynthetic rate , stomatal conductance, transpiration rate and Fv/Fm, as affected by drought stress at 5 and 10 days of interval in grafted okra genotypes R-NS7774/S-NS7772, R-NS77774/S-Green Gold and R-NS7774/S-OH3312, along with respective controls. Vertical bars indicate mean ± SE for n = 5. Means denoted by the different letter are significantly different at p ≤ 0.05 according to the Tukey’s studentized range test.

Our results illustrated that the use of NS7774 as rootstock notably increased all the gas exchange parameters and Fv/Fm (**Figure 5**). The net photosynthesis rate, stomatal conductance, transpiration rate and Fv/Fm for the homograft G1 notably increased by 4.2%, 11.4%, 10% and 9.8% respectively after 10 days of stress while for G2 and G3, all the parameters remained same and were maintained throughout 5 and 10 days of DS.

### Stomatal closure: A first line of defence against drought stress conditions and stomatal index

**Figure 6:**
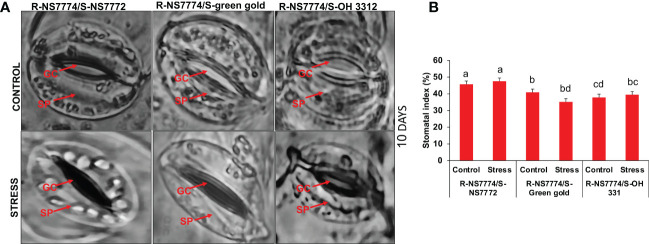
**(A)** Representative images of stomata, as affected by drought stress at 10 days of interval in grafted okra genotypes, R-NS7774/S-NS7772, R-NS77774/S-Green Gold and R-NS7774/S-OH3312 along with respective controls observed under dark field and phase contrast microscope at 40X magnification. In images, GC indicates guard cells and SP indicates stomatal pore. **(B)** stomatal index, as affected by drought stress at 5 and 10 days of interval in grafted okra genotypes, along with respective controls at 10X magnification. Vertical bars indicate mean ± SE for n = 5. Means denoted by the different letter are significantly different at p ≤ 0.05 according to the Tukey’s studentized range test.

In our study, it can be clearly seen that after 10 days of drought stress treatment the stomata were seen to be closed completely when compared to control for all the homograft genotypes (G1, G2 and G3) (**Figure 6A**). Among all the grafted okra genotypes under drought stress, G1 showed well organized guard cells with partially open stomata and the number of stomata was also observed to be higher as compared to other grafted okra genotypes under 10 days of drought stress (**Figure 6B**).

### Effects of drought stress on hydrogen peroxide, MDA content and electrolyte leakage of grafted okra genotypes

**Figure 7:**
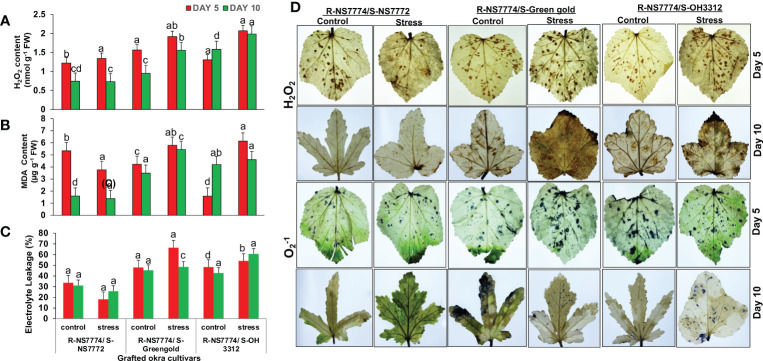
Changes in **(A)** H_2_O_2_ content, **(B)** Malanoaldehyde content, and **(C)** Electrolyte leakage (%) as affected by drought stress at 5 and 10 days of interval in grafted okra genotypes R-NS7774/S-NS7772, R-NS77774/S-Green Gold and R-NS7774/S-OH3312, along with respective controls. Vertical bars indicate mean ± SE for n = 5. Means denoted by the different letter are significantly different at p ≤ 0.05 according to the Tukey’s studentized range test **(D)** Histochemical localization of oxidative stress markers H2O2 and O2−, as affected by drought stress at 5 and 10 days of interval in grafted okra genotypes R-NS7774/S-NS7772, R-NS77774/S-Green Gold and R-NS7774/S-OH3312, along with respective controls. The brownish colour on the leaves indicates the localization of H_2_O_2_ stress marker, whereas the bluish colour indicates the localization of O2−1 marker.

After 5 days of drought stress treatments, the H_2_O_2_ content was found to have increased in G1, G2 and G3 respectively under drought stress when compared to control (**Figure 7A**). However, by increasing the exposure time to drought stress for 10 days, grafted genotype G1 showed reduced and lesser extent of H_2_O_2_ activity under stress treatment when compared to G2 and G3. In case of MDA content, the levels were seen to be increased in drought treatments for G2 and G3 while reduced for G1 by 29% after 5 days of drought stress treatment when compared to control (**Figure 7B**). However, after 10 days of stress, the level of lipid peroxidation reduced for homograft G1 more efficiently. Drought stress induced oxidative stress leads to membrane damage which is the main reason for increase in electrolyte leakage in plants (Assaha et al., 2016). Moreover, grafting showed a significant increase in membrane stability, and decreased the electrolyte leakage under drought stress. In our study we observed the same trend as we observed above for MDA content where the extent of electrolyte leakage was reduced for grafted plant (G1), for 5 and 10 DAS by 46 and 16% respectively when compared to G2 and G3 (**Figure 7C**). However, overall damage to grafted okra genotypes due the ROS was comparatively lesser when compared to non-grafted okra genotypes as examined in our previous study^22^.

### Grafting alleviated oxidative stress under drought stress

In our present study we investigated ROS formation in grafted okra genotypes using DAB staining for H_2_O_2_ localization and NBT staining for ${\text{O}}_{2}^{\;-1}$ localization (**Figure 7D**) where the accumulation of brown colour spots resemble H_2_O_2_ localization and blue spots resemble ${\text{O}}_{2}^{\;-1}$ localization. Less to no accumulation of brown and blue spots were observed on the surface of leaves under 10 days of drought stress for grafted genotype G1. However, appearance of brown and blue colour was observed for G2 and G3 under drought stress to a lesser extent under drought stress when compared to non-grafted okra genotypes as discovered previously (Razi et al., 2021). However, grafting could scavenge/neutralize the free radical formed, as is evident by the reduction of brown and blue spots in treatment groups G1, G2 and G3 when compared to their controls.

### Determination of proline content, total soluble sugars and protein content

**Figure 8:**
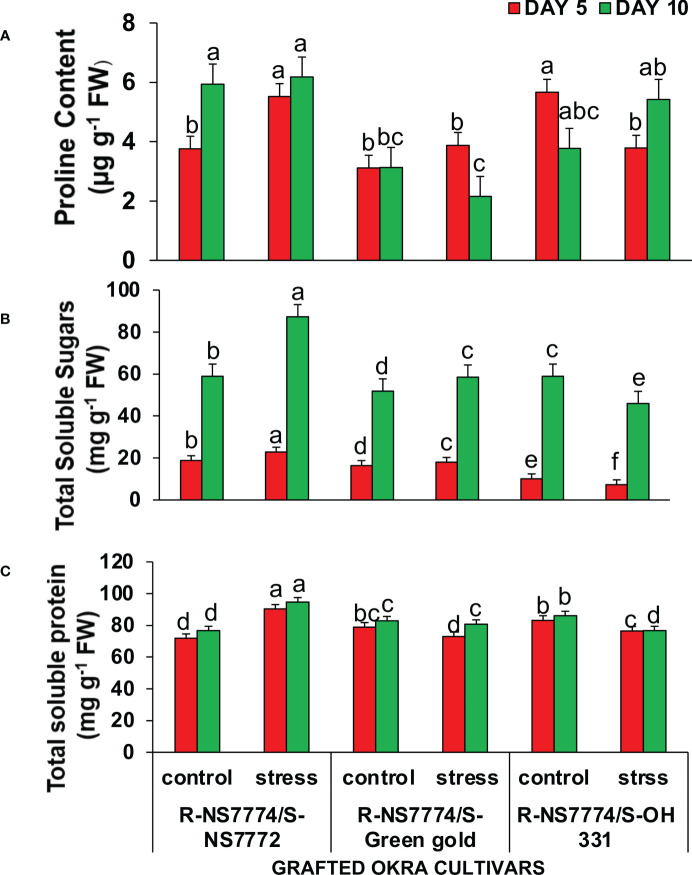
Changes in **(A)** proline content, **(B)** Total soluble sugar content, and **(C)** Total soluble protein as affected by drought stress at 5 and 10 days of interval in grafted okra genotypes R-NS7774/S-NS7772, R-NS77774/S-Green Gold and R-NS7774/S-OH3312, along with respective controls. Vertical bars indicate mean ± SE for n = 5. Means denoted by the different letter are significantly different at p ≤ 0.05 according to the Tukey’s studentized range test.

Total soluble sugar and total protein contents are crucial energy sources, especially under drought stresses. The results indicated that homograft genotypes G1, G2 and G3 contained a higher amount of proline, TSS and TSP under drought stress treatment compared with the control for 5 and 10 days of drought stress. However, NS7772 when grafted to rootstock NS7774 (G1) showed the highest amount of all the above mentioned osmolytes (**Figures 8A–C**)

### Antioxidant enzyme activity and their isozymes

**Figure 9:**
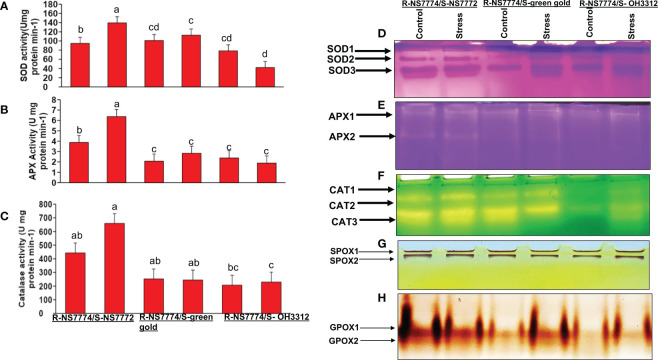
Changes in antioxidant enzyme activity and isozyme profiles of: **(A, D)** Superoxide activity (SOD), **(B, E)** ascorbate peroxidase (APX) and **(C, F)** catalase activity (CAT) activity, as affected by drought stress after 10 days of interval in grafted okra genotypes R-NS7774/S-NS7772, R-NS77774/S-Green Gold and R-NS7774/S-OH3312, along with respective controls. Vertical bars indicate mean ± SE for n = 5. Means denoted by the different letter are significantly different at p ≤ 0.05 according to the Tukey’s studentized range test. Non-denaturing activity gels were prepared and run as described in the method. The numbers in gels, indicate each isozyme of antioxidant enzymes in order of detected bands from the top. Profiles of peroxidase isozymes **(G)** SPOX **(H)** GPOX of grafted okra genotypes R-NS7774/S-NS7772, R-NS77774/S-Green Gold and R-NS7774/S-OH3312, for a period of 10 days. Non-denaturing activity gels were prepared and run as described in the method. The numbers in gels, indicate each isozyme of antioxidant enzymes in order of detected bands from the top.

ROS formation was confirmed by the above performed H_2_O_2_ and ${\text{O}}_{2}^{\;-1}$ localization in grafted okra leaves experiments under drought stress. To explore the detoxification of oxidative damage, three essential enzyme activities along with their isozyme expression were assessed. The activities of antioxidant enzymes that play a crucial role in ROS homeostasis in plants, such as superoxide dismutase (SOD), ascorbate peroxidase (APX), and catalase (CAT), were modified differentially under drought stress condition (**Figures 9A–C**). A significant increase in all three antioxidant enzymes activity was seen for all the grafted okra genotypes under drought stress in comparison to control plants. Antioxidant enzyme activities (SOD, APX, and CAT) were validated using isozymes expression analysis (**Figures 9D–F**) which demonstrated a similar pattern to the SOD, APX, and CAT expression patterns as seen by isozymes profile. The expression of isozyme bands of SOD was seen to be more intense in treatments groups where drought stress was provided for 10 days (**Figure 9D**). Three isozymes of SOD were actively stained, amongst which SOD-3 showed higher band intensities in G1, G2 and G3 under drought stress as compared to their control. Isozyme bands of SOD-2 was also seen to be expressed more in G1 under stress as well as control as compared to G2 and G3. The APX activity (**Figure 9B**) was significantly increased in G1 grafted genotype when compared to G2 and G3 which was validated by the two isozymes of APX as shown in **Figure 9E**. Out of two isozymes of APX that were stained, band intensity of isozyme APX 2 was increased in drought stress treated grafted okra genotypes G1 than G2 and G3 (**Figure 9E**). The CAT activity was seen to follow the same trend as seen in case of SOD and APX activity. Under 10 days of drought stress, activity of CAT was seen to have increased in G1 when compared G2 and G3 respectively (**Figure 9C**). Three CAT isozymes were stained, amongst which (**Figure 9F**) CAT-2 and CAT-3 isozyme bands showed higher band intensity in G1 and was less expressed in G2 and G3 under drought stress, respectively.

### Effect of drought stress on the isozymes of peroxidase enzymes

Changes in isozyme profiles of peroxidase enzymes GPOX and SPOX were observed. SPOX isozymes did not display much changes in expression level amongst the drought treated groups and control groups (**Figure 9G**). However, for G1, band intensity of SPOX-2 was seen to be higher when compared to G2 and G3 under drought stress. The active staining of GPOX revealed two isozymes GPOX-1, GPOX-2, amongst which the expression pattern of the bands was much intense for homograft G1 and no significant difference were observed for G2 and G3 in their control and stress group (**Figure 9H**).

### Identification of differentially expressed proteins in different comparison combinations and functional annotation of the identified proteins using gene ontology

**Figure 10:**
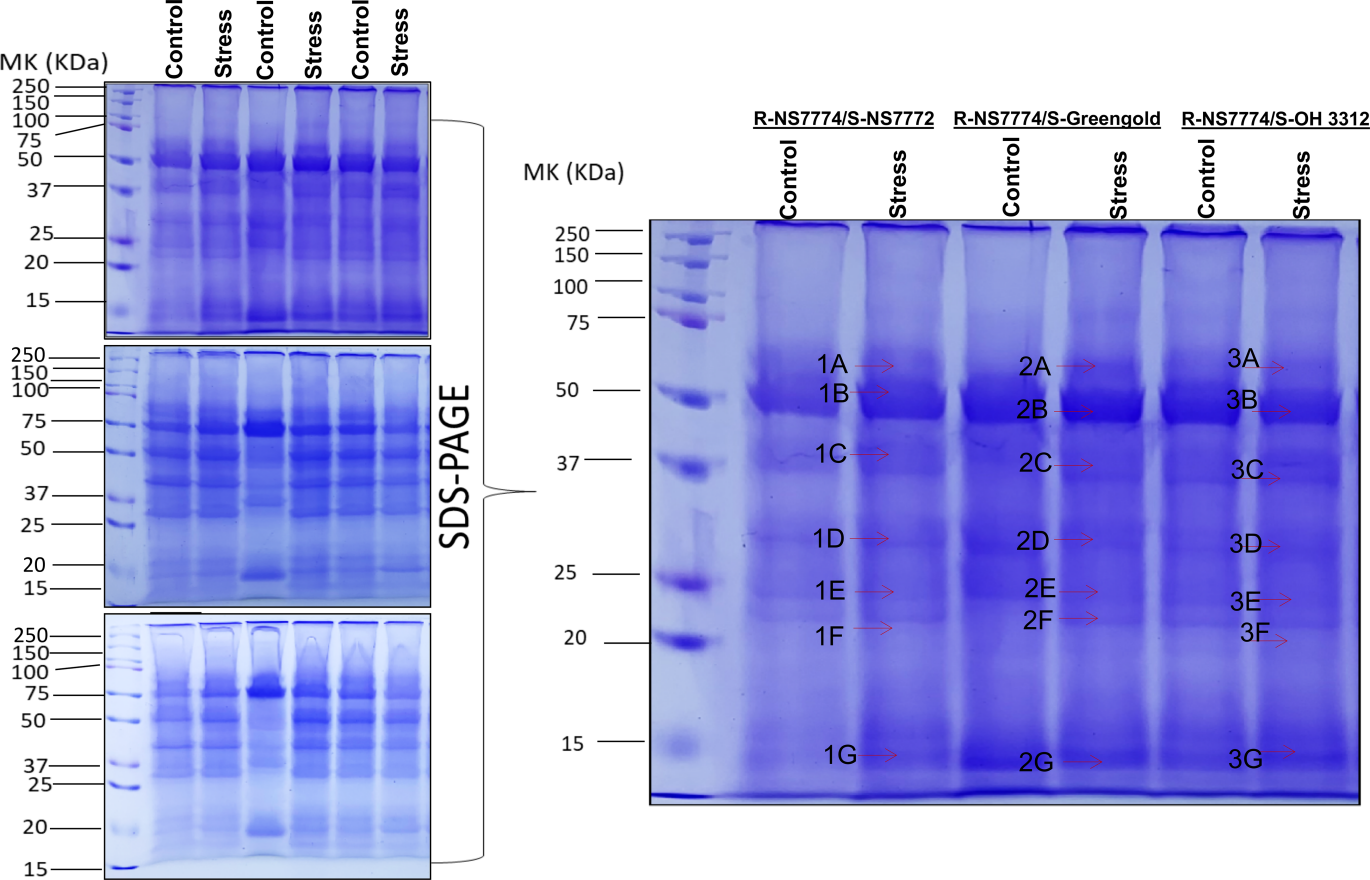
Representative image of protein profiles (SDS-PAGE) of grafted okra genotypes R-NS7774/S-NS7772, R-NS77774/S-Green Gold and R-NS7774/S-OH3312 for a period of 10 days. Differentially expressed bands excised for protein identification by LC-MS/MS are marked by arrows.

**Figure 11:**
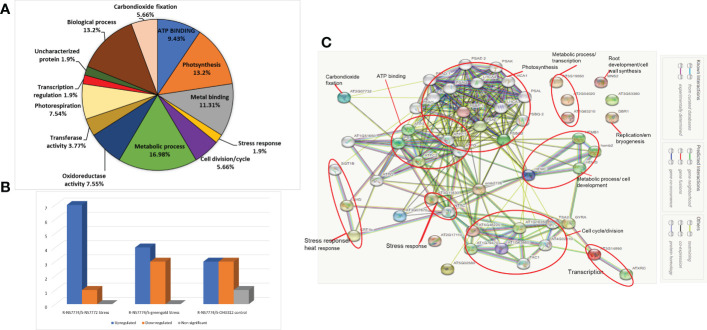
Comparative analysis of proteome profiles between the treatments. **(A)** Functional classification of identified protein by Gene ontology analysis, **(B)** Venn diagram illustration of up-, down- or non-significantly regulated proteins and **(C)** Analysis of protein interaction network by STRING 9.1.

First dimensional gel electrophoresis (SDS-PAGE) was used to analyse the protein profile and study about the upregulation and downregulation of protein (**Figure 10**). These up-or down regulated proteins were thereafter identified by mass spectrometer (LC-MS/MS) (**Table 1**). The identified proteins were then further categorized functionally in different groups using gene ontology (**Figure 11A**). The up-, down- or non-significantly regulated proteins were illustrated using Venn diagram (**Figure 11B**). All the differentially expressed proteins were grouped into photosynthesis (13.2%), metabolic process (16.98 %), oxidoreductase activity (7.55%), stress response (1.9%), and transcription regulation (1.9 %), Transferase activity (3.77%), Metal binding (11.31%), ATP Binding (9.43%), carbon dioxide fixation (5.66%), biological process (13.2%), cell division/cycle (5.66%), photorespiration (7.54%) and uncharacterized protein (1.9%) (**Figure 11A**). Further, the identified proteins were checked for any protein-protein interactions using STRING data base (**Figure 11C**).

**Table 1:** Identification of proteins analyzed by LC-MS/ MS.

Band no.	Protein name	Plant species	Protein ID	Protein score	Biological function	Mr value (expt)	Mr value (calc)
1A	Leucine--tRNA ligase	*Deinococcus radiodurans*	SYLDEIRA	30	ATP binding, Translation	1464.7772	1465.5748
1B	Ribulose bisphosphate carboxylase large chain	*Ipomoea purpurea*	RBLIPOPU	83	Photosynthesis, metal binding, photorespiration, oxidoreductase activity, metabolic process, biological process	804.5353	804.4745
1C	Ribulose bisphosphate carboxylase/oxygenase activase B, chloroplastic	*Hordeum vulgare*	RCABHORVU	29	ATP binding	2088.1881	2088.1619
1D	Oxygen-evolving enhancer protein 1, chloroplastic	*Spinacia oleracea *	PSBOSPIOL	47	Photosynthesis, biological process	2283.3171	2283.1535
1E	Oxygen-evolving enhancer protein 2, chloroplastic	*Fritillaria agrestis *	PSBPFRIAG	47	Photosynthesis, metal binding,	1172.6161	1172.619
1F	Ribulose bisphosphate carboxylase small subunit, chloroplastic 2	*Nicotiana sylvestris*	RBS2_NICSY	44	Photosynthesis	932.5598	932.508
1G	Ribulose bisphosphate carboxylase small subunit, chloroplastic	*Capsicum annuum *	RBSCAPAN	44	Metabolic process, photosynthesis, biosynthesis process, photorespiration, carbon dioxide fixation	932.5598	932.508
2A	Baruol synthase	*Arabidopsis thaliana*	BARS1_ARATH	16	Translation regulator activity	1405.8553	1405.6952
2B	Ribulose bisphosphate carboxylase large chain	*Adoxa moschatellina *	RBLADOMO	359	Oxidoreductase activity, Photosynthesis, metabolic process, metal binding, photorespiration, carbondioxide fixation	909.4109	909.4378
2C	Ribulose bisphosphate carboxylase/oxygenase activase B, chloroplastic	*Hordeum vulgare*	RCABHORVU	48	ATP binding	2088.3021	2088.1619
2D	Putative linoleate 9S-lipoxygenase 3	*Oryza sativa*	LOX3_ORYSJ	11	Biological process, Metal binding, oxidoreductase activity	841.5596	842.4187
2E	Spindle and kinetochore-associated protein 1 homolog	*Arabidopsis thaliana*	SKA1_ARATH	18	Cell cycle/ division	1172.7337	1172.6262
2F	DNA gyrase subunit A	*Chlamydia trachomatis *	GYRACHLTR	25	ATP binding	961.4731	960.524
2G	Ribulose bisphosphate carboxylase small subunit, chloroplastic	*Malus sp.*	RBSMALSP	43	Photosynthesis, metabolic process, biological process, photorespiration, carbondioxide fixation	932.5488	932.508
3A	GDSL esterase/lipase At2g04020	*Arabidopsis thaliana*	GDL33ARATH	21	Metabolic process, biological process	1464.7651	1464.7507
3B	ATP synthase subunit beta, chloroplastic	*Acrocomia aculeata*	ATPBACRAL	200	Metabolic process, biosynthesis process, cellular process, ATP binding	1327.7256	1327.6633
3C	Pentatricopeptide repeat-containing protein At3g53360, mitochondrial	*Arabidopsis thaliana *	PP280ARATH	22	Metabolic process, biological process, transcription	1134.71	1134.6001
3D	Putative membrane protein insertion efficiency factor	*Staphylococcus epidermidis*	YIDDSTAEQ	11	NIL	991.5114	992.4424
3E	Serine/arginine-rich splicing factor RSZ21A	*Oryza sativa subsp. japonica*	RZ21AORYSJ	16	Biological process, Metal binding, Metabolic process	1498.6109	1498.6437
3F	Cytochrome b-c1 complex subunit Rieske, mitochondrial	*Zea mays*	UCRI_MAIZE	18	Response to heat, Metal binding, oxidoreductase activity	1178.6518	1178.5932
3G	Achilleol B synthase	*Oryza sativa subsp. japonica*	ACBSYORYSJ	30	Metabolic process, biological process, cellular process	2096.2458	2096.0282

### Protein-protein interactions

The protein–protein interaction network generated using STRING 11.5 discovered functional relationship and interaction among different identified proteins using LCMS/MS for grafted okra genotypes under drought stress (**Figure 11C**). The key clusters of protein–protein interaction is highlighted in red coloured circles. PPI network generated helped to demonstrate the major group of proteins that were associated with photosynthesis, metabolic process, ATP binding, stress response, cell cycle and development. The STRING analysis of grafted okra genotypes exceptionally showed a higher interaction of plant photosynthesis proteins, than other functional categories of identified proteins. The protein–protein interaction results also depict a greater percentage of proteins related to stress response and metabolic process which could help in cell division and growth of plants even under drought stress conditions.

### Quantitative real-time RT-PCR analysis

**Figure 12:**
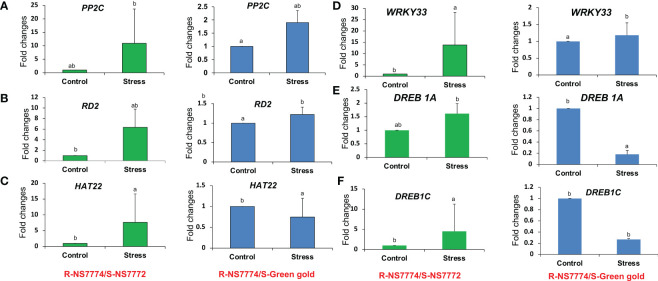
The relative expression level of drought stress responsive genes, **(A)** RD2 **(B)** HAT22 **(C)** PP2C **(D)** DREB1A **(E)** DREB1C **(F)** WRKY33 genes of grafted okra genotypes R-NS7774/S-NS7772, R-NS77774/S-Green Gold and R-NS7774/S-OH3312 for a period of 10 days. Vertical bars indicate Mean±SE of the means for n = 3. Means denoted by the different letter are significantly different at P≤0.05 according to the Tukey’s studentized range test.

To confirm our findings, we conducted a supporting experiment by using quantitative real-time PCR (qRT-PCR). We made the selection of genes based on the following criteria: highly differentiated in response to drought stress and reported to be potentially associated with drought tolerance. The expression of *PP2C*, *RD2*, *HAT22*, *WRKY33*, *DREB1A* and *DREB1C* genes was increased in grafted okra genotypes NS7772 and green gold under 10 days of drought stress as compared to control (**Figures 12A–F**). For the genotype green gold, reduced expression was observed exceptionally for *DREB1A* and *DREB1C* alone, but overall, the increased expression for other genes were crucially responsible for drought tolerance after grafting. The grafted genotype NS7772 obviously showed the highest expression levels under drought stress indicating efficient drought tolerance.

### Yield study

Grafted Okra genotypes exhibit variability with respect to various growth and yield traits (**Table 2**). The yield parameters including fruit diameter (mm), plant height (cm), stem diameter (mm), total number of fruits, length of pod (cm), number of seeds per pod and total yield. Maximum fruit diameter was recorded for grafted okra genotype NS7772 (17.9 mm) followed by grafted OH3312 (16.12mm) and then grafted green gold (15.57mm). The shoot height varied from 150 cm to 160 cm, the maximum was recorded for G1 (161cms), and the minimum for G2 (151 cm). Similarly, maximum stem diameter was recorded for G1 that is 8.2mm followed by G2 (7.1mm), and then G3 (6.9mm). Total number of fruits also varied with maximum for G1 (75), followed by G3 (55) and then for G2 (49). Length of the pods also varied among the grafted okra genotypes with maximum length for G1 (14.68 cm), followed by G3 (11.51cm) and then G2 (11.01cm). The maximum number of seeds per pods was observed for G1 (61) followed by G3 (53), and the minimum number was observed for G2 (47). Similar results were observed for fruit yield with maximum yield for G1 (1520.6 g), followed by G3 (1274 g), and minimum yield out of all was observed for G2 (1223.7 g).

**Table 2:** Yield parameters of grafted okra genotypes R-NS7774/S-NS7772, R-NS77774/S-Green Gold and R-NS7774/S-OH3312 after 75 days of grafting.

	R- NS7774 / S-NS7772	R-NS7774 / S-green gold	R-NS7774 / S-OH 3312
**Fruit diameter(mm)**	17.9^a^	15.57^c^	16.12^b^
**Shoot height (cm)**	161^a^	151^c^	155^b^
**Stem diameter (mm)**	8.2^a^	7.1^b^	6.9^bc^
**Total No of fruits**	75^a^	49^cd^	5 5^b^
**Length of pod (cm)**	14.68^a^	11.01^b^	11.51^b^
**No of seeds per pod**	61^a^	47^b^	53^b^
**Total yield (g)**	1520.6^a^	1223.7^c^	1274^b^

^a-d^ Represents statistical significance.

## Discussion

In water-restricted environments, drought is the main factor limiting okra productivity. The use of drought-resistant genotypes is recommended because it reduces the negative impacts of drought stress, increases yield, and has positive effects on the environment, the economy, and society (Mukhtar et al., 2014). Grafting expedites the incorporation of genetic resistance into the cropping system compared to the production of new genotypes, which is advantageous because drought stress is an urgent and widespread issue (Pofu et al., 2012). Although grafting vegetables is a centuries-old tradition, it wasn't until the 20th century that ornamental and herbaceous vegetables were improved by grafting (Lee et al., 2010). Since then, this approach has been used significantly more often in ornamental cactus, watermelon, melon, cucumber, eggplant, pepper, and tomato, but have not been exploited in okra (Trinchera et al., 2013). This is mostly due to the unavailability of rootstock genotypes for okra that are both resilient to biotic and abiotic stresses and capable of increasing commercial yields to balance the additional expenses associated with grafting. Due to the robust root system utilised as the rootstock, grafted plants typically exhibit enhanced uptake of water and nutrients compared to non-grafted plants (Sánchez-Rodríguez et al., 2013). In the present study, three different locally grown okra genotypes NS7772, green gold and OH3312 (*Abelmoschus esculentus* L.) were selected as scions (drought sensitive genotypes as proved from our previous study) grafted onto one rootstock (NS7774) having potential to increase the tolerance to water stress (Razi et al., 2021). We could observe that under well-watered conditions and stress treatment, grafted genotypes (G1, G2 and G3) thrived better based on plant (shoot) height, root length, biomass (fresh and dry weight) except for homograft G1, which showed maximum tolerance towards drought stress in terms of increased biomass, increased photosynthesis and RWC content, lower lipid peroxidation and greater osmotic activity in leaves compared to the non-grafted okra genotypes as observed previously (Razi et al., 2021). From **Figures 1A, B**, clearly it can be observed the successful graft union formation and further the growth of grafted plants by their morphological appearance under control and drought stress situation and how grafting have helped in diminishing the damaging effects of drought stress by observing the healthy phenotype of grafted plants. Notably, in this work, we discovered that plants grafted with drought-tolerant okra rootstock showed an excellent approach to cope with water shortage for 10 days, as evidenced by the high level of biomass (**Figure 2**) accumulation and the maintenance of good water status in the leaves under drought stress (**Figure 3B**). These findings are consistent with previous work (Liu et al., 2016), where it was observed that cucumber plants showed higher tolerance levels for drought, as seen by enhanced biomass accumulation under drought stress, when drought-resistant luffa was used as a rootstock. It is of known fact that the primary organs of plants for absorbing water are their roots, which also play a key role in their resilience to drought stress. Root systems can regulate their own growth, development, water absorption, and transport to respond to drought stress (López-Marín et al., 2017). In this study, NS7774 rootstock specifically retained its greater root structure, root length, and biomass in the graft combination with NS7772 scion, leading to increased LRWC under water stress. Under mild and severe drought stress, homograft (G1) root length proliferation was observed (**Figure 3A**), which was advantageous for the root system to absorb the deep soil water and boost usage rate, thus improving the drought tolerance of the plant. The RWC of the grafted plants was calculated for drought stress periods of 5 and 10 days. Water status is a crucial plant physiological indicator, and leaf RWC reveals the severity of drought-like symptoms and the effectiveness of water absorption (Anjum et al., 2011). Under drought stress, we found that all genotypes of grafted okra plants had increased leaf RWC (**Figure 3B**). This implied that the grafted plants developed greater water use and transport capacity. Out of all the groups, G1 exhibited a marginally higher RWC. This marginal improvement in RWC in the leaves found after 10 days of drought stress may have been caused by osmotic adjustment as a result of the accumulation of proline, sucrose, and protein that was seen in this study.

A decrease in chlorophyll content is due to stomatal closure, which reduces CO_2_ uptake, and ion toxicity in chloroplasts, Chlorophyll degradation by ROS and due to activation of the chlorophyllase enzyme (Saha et al., 2010). The maintenance of chlorophyll content (Poór et al., 2011) is regarded as one of the essential aspects of a plant's drought resistance. In rootstock-grafted plants, the values of photosynthetic pigments (chlorophyll content and carotenoid content) were higher for G1 alone under drought stress (**Figures 4A, B**), indicating that rootstock grafting helped to minimise the drought-induced suppression of photosynthesis. According to the previous work (Abdellaoui et al., 2018), grafted tomato plants were much more chlorophyll-rich when subjected to heat stress. Carotenoids serve an important role in the heat dissipation of excess excitation energy in the photosynthetic machinery, which helps to prevent the first formation of superoxide in plants however; photo-protection of the photosystem by carotenoids may have played a vital role in controlling ROS production in chloroplasts.

The effect of drought stress on vascular transport activity in grafted okra plants has not been previously studied; however, our results imply that grafting may facilitate transport of minerals and ions from rootstocks to scions appropriately. Vascular transport activity has been previously studied in many grafted plants, including sweet cherry (Olmstead et al., 2006) and watermelons (Muneer et al., 2015), this is the first time that we have studied for grafted okra genotypes under drought stress. The stained portion between the rootstock and scion of grafted okra genotypes showed suitable and effective connection among vessels and the efficient functioning of transport of nutrients and other essential minerals (**Figure 4C**).

When there is a water shortage, plants quickly respond by closing their stomata to avoid water loss through transpiration. This action also limits their ability to absorb carbon dioxide, which lowers photosynthetic assimilation, which in turn reduces vegetative growth and yield (Grant, 2012). According to our previous study (Razi et al., 2021), we observed decrease in the measured photosynthetic parameters for non-grafted okra plants, which is in contrast to our present study where enhancement in the above indices in the grafted okra genotypes under drought stress was observed than control (G1, G2 and G3) which could primarily be attributed to these plants having higher Gs, Tr, and WUE (**Figure 5**). These outcomes were in line with other studies on apple plants grafted onto several rootstocks with varying drought tolerance (López-Marín et al., 2017). These findings demonstrated that grafting onto rootstocks could improve the drought tolerance of okra, because of the decreased incidence of stomatal or non-stomatal factors that inhibit photosynthesis and higher photosynthesis pigments (Zheng et al., 2016). The maximal PSII photochemical efficiency (Fv/Fm) is frequently employed as an indicator of PSII photo inhibition or stress damage (Cazzola et al., 2020). In our research, F_v_/F_m_ reduced with prolonged water stress for G2 and G3, but the values in the homograft G1 plants under drought circumstances were higher than in other plants as compared to their control.

The stomata often close in conditions of water stress to stop excessive water loss from the leaves and lessen transpiration. According to Cornic and Fresneau, 2002, drought stress mostly affects photosynthesis by reducing/closing the stomatal aperture. In the current study, plants grafted onto the rootstock had partially opened stomatal apertures, indicating that these plants are capable of sustaining stomatal opening and high photosynthetic efficiency even in drought-prone environments. Reduced leaf stomatal aperture (W/L) and density resulted in enhanced growth and a higher survival rate of the grafted okra plants under drought stress due to reduced transpiration water loss and improved water utilisation. The modulation of stomatal behaviour is essential for increasing plant tolerance to abiotic stress. Our results are in correlation to the previous studied reported (Kim et al., 2006) and hence proves how grafting is more efficient in stomatal structure and opening and closing of stomata. The number of stomata increased more in grafted plants under drought stress with maximum number of stomata in grafted okra genotype G1. (**Figures 6A, B**).

There is accumulation of reactive oxygen species (ROS), when plants are exposed to abiotic or biotic stress, such as drought stress, pathogen attack, high salt stress, chilling stress, or metal toxicities (Muneer et al., 2012). H_2_O_2_ was detected in the grafted plants under drought stress to a reduced amount compared to the control (**Figure 7A**). This effect was seen in cucumber plants grafted onto fig leaf gourds (*Cucumis ficifolia*) or onto luffas (*Luffa cylindrical*), where a lower H_2_O_2_ concentration at high temperatures reduced membrane lipid peroxidation (Das et al., 2015). The scientists attributed this decrease to higher ROS scavenging and CO_2_ assimilation. The plasma membrane is impaired by drought stress, which is indicated by an increase in plasma membrane permeability and partial electrolyte leakage. The presence of MDA and alterations in the permeability of the plasma membrane are crucial markers of the severity of the damage to the membrane. Surprisingly, this study revealed that the grafted plants had lower MDA content and relative electrical conductivity than the non-grafted okra plants, indicating that they were better able to withstand droughts (**Figures 7B, C**). H_2_O_2_ and ${\text{O}}_{2}^{\;-1}$ being one of the major ROS also act as vital signal molecules under drought stress (Chaves et al., 2003). There is always a homeostasis in ROS formation and its scavenging with an appropriate accumulation of antioxidant enzymes and other molecules located in various cell compartments of plants; however, when plants are exposed to stress situation for a prolonged period of time, drought stress will inevitably result in oxidative damage due to the over production of reactive oxygen species, as shown in previous studies (Bai et al., 2011). In this study, ROS were produced mostly on the surface of leaves of grafted okra plants under drought stress (**Figure 7D**). The oxidative stress was, however, greatly reduced by grafting as seen in our result. Our findings were validated by prior studies on stressed plants like *Capsicum annuum* (Soundararajan et al., 2017) and *Lycopersicon esculentum* (Muneer and Jeong, 2015). Plants actively accumulate osmotic regulatory substances (proline, sucrose, and protein) under drought stress to increase the concentration of cell fluid, which has the primary purpose of maintaining cell turgor, balancing the infiltration of protoplasm and the external environment, and allowing various physiological processes of cells to proceed normally (Zhang et al., 2017). Under drought stress, particularly after 10 days of stress, the amounts of soluble sugars, proline and protein in the leaves of grafted okra genotypes considerably increased (**Figures 8A–C**). The findings of this study are comparable to those of Guo's et al., 2017, which also demonstrated that the content of osmotic regulatory compounds like proline and soluble sugars increased significantly under drought situations as the stress duration increased. These soluble sugars have crucial roles as signalling molecules, controlling biosynthesis and sensing plant hormones. They also serve as osmotic regulators, preserve macromolecules (such proteins) and membranes, and provide fuel carbon for energetic metabolism when sunlight is decreased (Rolland et al., 2006). Similar findings have been reported for other plant species under water stress, including *Arabidopsis thaliana* (Sperdouli and Moustakas, 2012), and *Capsicum annuum* (Anjum et al., 2012) and they may reflect an adaptive response to the imposed stress. The increase in proline concentration values in okra leaves in dependency on the time and drought level is in accordance with these findings. Since proline can function as both an osmotic agent and a radical scavenger, proline accumulation has been linked to a plant's ability to withstand drought stress (Kauer and Asthir, 2015). Proline may also function as a metal chelator and a free radical scavenger, shielding leaves from lipid peroxidation, which occurred in okra under drought stress as previously mentioned (Gill and Tuteja, 2010). The greater levels of proline that were observed in plants under severe and moderate stress conditions may have been crucial for the recovery of the plants after stress.

With the help of enzymes like SOD, CAT, and APX, plants have developed an effective antioxidant system that prevents oxidative damage brought on by accumulated ROS. The expression levels of the antioxidant enzyme genes APX, SOD, and CAT in the leaves of the drought-tolerant grafted genotypes G1, G2, and G3 were greater, which is consistent with the results of the enzyme activity assay, while the highest expression was found for G1 under drought stress (**Figures 9A–C**). These ROS scavengers are often water soluble, and they are eliminated during ROS detoxification or through self-oxidation, which may account for the modest drop-in activity. During an extended period of stress, the cell's capacity to resynthesize the damaged or oxidised scavengers is limited. As a result, while under sustained stress, tissues are especially vulnerable to ROS attack. In our study, the antioxidative enzyme activity and their isozyme expression profile are significantly correlated (**Figures 9D–F**). The significant positive association between enzyme activities and related isozyme expression raises the possibility that the transcription levels of antioxidant isozymes have an impact on the antioxidative capabilities of grafted okra leaves. Numerous additional crops, including tomato (Zhang et al., 2019), have been grafted with tolerant rootstocks to confer effective protection against ROS.

Yet another defence mechanism adopted by plants under drought stress is lignification which is accomplished *via* the activities of ionically or covalently attached cell wall peroxidases (Boudet, 2000) enzymes such as GPOX, SPOX and PPO that play a role in the anti-oxidative cycle. In this work, the observed rise in GPOX and SPOX (**Figures 9G, H**) was substantially linked with the increase in lignin content after 10 days of water deficit. According to Boudet, 2000, lignification is catalysed by the oxidative polymerization of monolignols such as coumaryl, coniferyl, and sinapyl alcohol. when H_2_O_2_ is present the observed increases in peroxidase activity may be due to changes in the cell wall's mechanical characteristics, which can be linked to adaptation to drought. The increased peroxidase activity could alter the structure of cell wall, which in turn could be correlated with adaptation to drought stress (Cervilla et al., 2009).

To repair the oxidative damage induced by drought stress, plants reprogram their transcriptome, proteome, and metabolome, thereby modifying the expression of many transcripts, proteins, metabolites, and lipids. Our physiological findings indicate that rootstocks and scions exhibited considerable drought stress induced alterations. Previous research, (Zheng et al., 2010) focused mostly on the physiological and biochemical responses of grafted plants. With the exception of our recent discovery on the proteome in okra grafted plants, there has been no investigation on protein expression in abiotically stressed grafted okra plants under drought stress. We used one-dimensional gel electrophoresis (**Figure 10**) followed by mass spectrometry to analyse proteins in grafted okra genotypes under drought stress. Using LC/MS-MS, the differentially expressed proteins from grafted plants were extracted from 1-DE gels and analysed (Muneer et al., 2015). These findings provide an overview of the proteins involved in graft unions as well as novel insight into acclimation to drought stress. Using gene ontology analysis (www.geneontology.com), we categorised over all 21 proteins from the grafted okra genotypes G1, G2 and G3 into distinct functional categories (**Figure 11A**). The identified proteins were functionally classified into groups, such as photosynthesis (13.2%), metabolic process (16.98%), transcription (1.9%), stress response (1.9%), ATP binding (9.43%), Metal binding (11.31%), oxidoreductase activity (7.55%), photorespiration (7.54%), biological process (13.2%) etc.

### Proteins related to photosynthesis

Photosynthesis is an essential plant process and is sensitive to many environmental stresses including temperature and heat stress. Among all identified proteins in grafted okra genotypes, 13.2% of the proteins were classified into photosynthesis. In this study, the majority of Calvin-cycle-related proteins, including the Rubisco large subunit, Ribulose bisphosphate carboxylase small subunit, chloroplastic 2, Ribulose bisphosphate carboxylase small subunit, chloroplastic ,Ribulose bisphosphate carboxylase small subunit 1B chloroplastic, Oxygen-evolving enhancer protein 1 chloroplastic and Oxygen-evolving enhancer protein 2 were upregulated for the grafted okra genotypes under drought stress (Band 1B, 1F , 1G, 2B, 2G and 3B respectively of **Figure 10**). Ribulose-1,5-bisphosphate carboxylase/oxygenase (RuBisCO) is the essential enzyme for CO_2_ fixation during photosynthesis. In particular, the RuBisCO small subunit is required for carboxylation catalytic efficiency and CO_2_/O_2_ specificity (Krech et al., 2012). Higher RuBisCo protein accumulation in grafted plants enables photo protection and an enhancement in the light harvesting mechanism for plant physiological development (Yamori et al., 2016). Oxygen-evolving enhancer protein, a part of the oxygen evolving complex of PSII, is involved in the light reaction of PSII (Mayfield et al., 1987). The expression of OEE1 and OEE2 (Band 1D and 1E of **Figure 10**) in grafted okra genotypes increased under drought stress as compared to their control, but significant increase was observed for scion NS7772 when grafted onto NS7774 rootstock. These results indicated that NS7774 rootstock played a vital role in maintaining the stability of PSII, and are consistent with a previous proteomics study (Sang et al., 2017). However, the expression of OEE2 was different from OEE1, and the relationship between OEE1 and OEE2 is not clear yet. The upregulation of above-mentioned photosynthesis related proteins shows that grafted okra plants exhibit enhanced cell communication associated to photosynthetic activity under drought stress. The identification of photosynthetic proteins provides two essential confirmations: the prevalence of temperature anomalies for proper photosynthesis and the discovery of photosynthetic proteins in graft unions.

### Proteins involved in energy metabolism and metabolic processes

Adequate adenosine triphosphate (ATP) is essential for plant responses to abiotic stress (Hu et al., 2014). The stability and activity of ATP synthase are both important factors in the regulation of ATP synthase. The catalytic sites of ATP synthase are carried completely or primarily on the beta subunit of the enzyme (Lee et al., 1991). ATP synthase (Band 3B) was upregulated when grafted plants were grown under drought stress, and suggested a greater energy requirement for the degradation and biosynthesis of proteins (Das et al., 2015). Interestingly, ATP synthase was also up-regulated in plants grafted on drought tolerant okra rootstock, which indicated that the process of ATP biosynthesis was active (Rolletschek et al., 2011) indicating optimal solute synthesis and transport from source to sink in response to drought stress. Under drought stress, the protein GDSL esterase/lipase At2g04020 (Band 3A), an essential hydrolytic enzyme with multifunctional properties such as broad substrate specificity and region specificity was seen to upregulate the protein lipase. Activation of lipases (lipid hydrolysing proteins) that cleave or alter lipid substrates in many subcellular compartments facilitates the majority of lipid-associated plant defense responses. Furthermore, two Ribulose bisphosphate carboxylase/oxygenase activase B, chloroplastic proteins were induced by drought stress (Band 1C and 2C). Additionally, ATP synthesis related proteins including Leucine--tRNA ligase (Band 1A) and DNA gyrase subunit A (Band 2F) were altered for grafted okra genotype G1 and G2 under drought stress. Their changes imply that grafting enhanced energy production in okra genotypes to cope with moderate drought stress.

Pentatricopeptide repeat-containing protein At3g53360, mitochondrial (Band 3C) is an essential protein that is constantly involved in the progression of organelle transcript cleavage, stability, and translation, and functions as post-transcriptional editing factors (Okuda et al., 2007) also showed upregulation in the grafted okra genotype G3 in response to drought stress. During drought stress, a protein identified as Serine/arginine-rich splicing factor RSZ21A (Band 3E) was also elevated. SR (serine/arginine-rich) proteins are a highly conserved family of RNA-binding proteins that are essential for the execution and regulation of precursor-mRNA (pre-mRNA) splicing. Serine/arginine-rich proteins (SR) are fundamental regulators of constitutive and alternative pre-mRNA splicing, but recent studies reveal they also regulate mRNA export, stability, and translation (Howard and Sanford, 2015). The number of SR genes encoded by plants is greater, with 18 in A. thaliana, 25 in Brassica rapa, 22 in Oryza sativa, and 40 in Triticum aestivum (Chen et al., 2019).

In addition to these proteins, few proteins involved in other physiological processes were revealed in this study. For example, three proteins identified as Spindle and kinetochore-associated protein 1 homolog (Band 2E), ATP synthase subunit beta, chloroplastic (3B) and Achilleol B synthase (3G) were involved in the category of cell cycle/division. Spindle and kinetochore-associated protein 1 homolog (Band 2E), an important protein involved in cellular response was increased under drought stress situation. The upregulation of this protein enables microtubule binding activity, other cellular processes including chromosome segregation, mitotic cell cycle and regulation of microtubule polymerization or depolymerization. Thus, grafting mediates the efficient regulation of metabolic processes in the plants such that the plants can cope up with the abiotic stress.

### Proteins related to metal binding

Proteins involved in the transport or binding of ions play an essential role in plant vascular tissues. The vast number of ion transport/binding proteins in the vascular cambium of plants has been well characterised (Neilson et al., 2010). Upregulation of proteins related to ion binding, such as Putative linoleate 9S-lipoxygenase 3 (Band 2D), Serine/arginine-rich splicing factor RSZ21A (Band 3E) and Cytochrome b-c1 complex subunit Rieske, mitochondrial (Band 3F) were observed under drought stressed condition. In the realm of plant physiology, the activity of lipoxygenase (LOX) protein is regarded as a reliable biological marker (Pokotylo et al., 2015). The activity of this protein increases in plants subjected to drought stress, pathogen infection, and mechanical injury (Maccarrone et al., 2001). Enhanced LOX activity at low temperatures is associated with the action of phospholipase D, which leads to the breakdown of phospholipids and the release of PUFAs, which are LOX substrates. The increased number of these ion binding/transport proteins detected in the proteomic study of grafted okra plants (**Table 1**) suggests proper communication between the rootstock and scions. Under drought stress, grafted okra genotypes revealed up-regulation of ion transport/binding. This demonstrates that oxidative damage is incapable of disrupting the movement of minerals and ions through the vascular bundles of grafted okra genotypes. Nevertheless, graft unions may be able to survive stress conditions to a certain extent for optimal transport activity by boosting stress-responsive proteins, as demonstrated by our RT-PCR analysis (**Figure 12**) and our examination of the enzyme activities of stress-responsive proteins (**Figure 9**).

### Proteins related to transcription and translation

Protein synthesis performs crucial physiological roles in the response of plants to drought stress. The expression of genes that play a vital role in protein synthesis has been studied for various plants (Zhao et al., 2007). Our proteome data (**Table 1**) of grafted okra genotypes identified several differentially expressed proteins that are related to protein synthesis that includes Leucine--tRNA ligase (Band 1A), Baruol synthase (Band 2A) and Pentatricopeptide repeat-containing protein At3g53360, mitochondrial (Band 3C). The aminoacyl-tRNA synthetases (also known as aminoacyl-tRNA ligases) catalyse the attachment of an amino acid to its cognate transfer RNA molecule in a highly specific two-step reaction (Woese et al., 2000). The upregulation of proteins involved in protein synthesis in grafted okra plants shows the amplification of translational processes, and may be a result of ribosomal activity for proper protein synthesis during drought stress. In addition, our results discovered proteins associated to protein synthesis that had not before been seen in grafted okra plants (Yu et al., 2008).

### Proteins related to defense response

Stress-responsive proteins play a crucial function in the detoxification of a wide variety of abiotic stressor (Li et al., 2013). RT-PCR study validated the suppositional upregulation of proteins associated with stress resistance under drought stress (**Figure 12**). Cytochrome b-c1 complex subunit Rieske, mitochondrial (Band 3F) plays an important role in repairing oxidative damage. The 2‐Cys peroxiredoxins (2‐CP) were recently identified as members of the antioxidant defence system of chloroplasts which plays a role in cell protection against oxidative stress by detoxifying peroxides (Pulido et al., 2010). It catalyses the reduction of hydrogen peroxide and organic hydroperoxides to water and alcohols, respectively. The expression of these defense response proteins indicates subsequent detoxification of ROS in all grafted okra genotypes, either resistant or sensitive. Thus, the identification of stress/defense sensitive proteins strongly implicates a robust antioxidant pathway in drought-stressed okra plants that have been grafted and are hypothesized to play a role in healing during grafting (Muneer et al., 2015).

### Protein–protein interaction network

Proteins in plant cells and tissues do not function as isolated molecules, but rather play coordinated and interconnected roles within networks (Nouri et al., 2011). Using the String 10.5 database, further research was conducted on the identified proteins in order to comprehend how drought stress signals are sent *via* protein-protein interactions to alter specific biological processes in grafted okra leaf cells. Six sets of interacting proteins were identified (**Figure 11C**). The first and largest network consists of photosynthetic machinery-regulating proteins. This category includes Photosystem I reaction centre subunit IV B, chloroplastic (PSAE-2), Photosystem I reaction centre subunit (psak), Light-harvesting chlorophyll-protein complex I subunit A4 (LHCA4), -type h+-transporting ATPase subunit gamma; ATP synthase gamma chain 1 (ATPC1), etc. The second group comprised ATP3, ATPC2, ATPC1, and PB linkage. These proteins are essential for ATP binding because they produce ATP from ADP when there is a proton gradient across the membrane. Protein SGT1 homolog B (SGT1B), Chaperone protein htpG family protein (SHD), Calreticulin 1b (CRT1B), and 2-Cys peroxiredoxin BAS were implicated in the third interaction network (AT3G11630). These proteins are required for the maintenance of the antioxidant defence mechanism and are involved in plant innate immunity. Similarly, the fourth interaction network contains proteins that are essential to the cell cycle and division procedure. The fifth interaction network contains proteins engaged in many metabolic processes, such as peptide synthesis (HEMB1, HEMB2). These findings corroborate the significance of these metabolic pathways in the response to drought stress, as reported before (Zhang and Shi, 2018). Moreover, the few nodes that are not related to other proteins within the interaction networks (such as AT3G53360, DBR1, and MNS2) indicate that these proteins do not interact with others according to the findings of the String database. These results suggest that the previously described proteins may influence the drought tolerance of grafted okra by interaction with these proteins directly and indirectly.

Previous research has uncovered multiple genes that are responsive to drought stress in a wide variety of plant species. Among these genes, the response to desiccation protein-encoding RD2 was critical for drought tolerance in cotton (Hou et al., 2018). Similarly, *RD1* and *RD2* genes were reported to be up-regulated in rice during drought stress as a result of seed priming (Samota et al., 2017). Likewise, we also observed the same correlation for the gene *RD2* under drought stress in grafted okra plants (G1 and G2), where it was significantly overexpressed for grafted okra genotypes NS7772 and green gold under drought stress comparative to their control, with G1 exhibiting the highest expression for the gene *RD2* under drought stress in comparison to G2 (**Figure 12A**). In the presence of drought stress, the expression of *HAT22* mRNA increased in the grafted genotypes G1 and G2 compared to their control, but the expression of the gene was higher in G1 than in G2, indicating that G1 is a more drought-tolerant grafted okra plant (**Figure 12B**). Increased expression of this gene decreased leaf yellowing in grafted okra genotypes under drought stress (Bouaziz et al., 2012). *HAT22* regulated stress-related genes, such as abscisic acid, in response to drought stress. ABA Abscisic acid (ABA) is an essential plant hormone that controls plant growth and resilience to biotic and abiotic stressors. It controls transpirational water loss by controlling the opening and closing of stomatal pores in order to resist drought stress. Additionally, during drought stress, we found elevated *PP2C* expression in the grafted okra genotypes NS7772 and green gold, which is consistent with other studies showing that *PP2C*s are essential for regulating ABA Signalling and signal transduction pathways (Shan, 2004) (**Figure 12C**).

Some stress-related genes, such as dehydration-responsive element-binding proteins (DREBs), regulate signalling pathways and interact with cold- and dehydration-responsive elements (DREs) in plants to confer tolerance to freezing, drought, and salinity by controlling the relevant signalling pathway. *GhDREB1A* and *GhDREB1B* overexpression in transgenic tobacco increased resistance to low-temperature and high-salt stress (Rushton et al., 2010). This is consistent with our findings that, under drought stress conditions, the expression levels of *DREB1A* and *DREB1C* were significantly higher in the grafted genotype NS7772 than in the control (**Figures 12D, E**). This shows that grafting played a significant impact in drought tolerance by preserving the transcript levels of genes that respond to drought stress in grafted okra plants. Thus, altering the expression of just one DREB/CBF gene may control the expression of other TFs, which in turn may activate a number of downstream target genes, enabling plants to withstand stress.

WRKY transcription factors regulate plant activities such as biotic stress responses (Banerjee and Roychoudhury, 2015). Recently, their involvement in abiotic stress responses has been reported (Ren et al., 2010). Multiple WRKY transcription factors have been identified as ABA signalling components. *WRKY18* and *WRKY60* regulate ABA signalling positively during seed germination and stress response. The *WRKY63* gene has been implicated in drought responses in a recent study (Zhou et al., 2014). In addition to these WRKY genes, numerous others have been implicated in drought and salt stress responses *WRKY33* demonstrated specific expression in leaves and may be elevated by drought stress and ABA treatment, which is concordant with our results where we observed that, grafted okra genotypes showed increased *WRKY33* transcription factor (TF) expression (**Figure 12F**).

In our investigation, the grafted okra genotypes (G1, G2, and G3) differed considerably across all yield parameters (**Table 2**). During harvest, the height of grafted okra plants varied between 150 and 162 cm. G1 plants were the tallest (161 cm), followed by G3 and G2. This characteristic had considerable selection potential. This may be a result of the ability of grafted okra genotypes to withstand drought stress after grafting. However, the NS7772 grafted genotype demonstrated successful graft compatibility, since these plants had the maximum yield parameter values. Similar variances in stem diameter were detected, which could be attributed to changes in genetic structure and environmental factors. Reddy et al., 2013 have had similar outcomes. Maximum fruit length (cm) was observed for hybrid G1 (14.68cm), followed by hybrids G2 (11.51cm) and G3 (11.51cm) (11.01cm). The difference in average fruit length (cm) between hybrids may be due to their diverse genetic background. Singh et al., 2006 observed similar findings. G1 produced the biggest fruit circumference, followed by G3 and G2. The grafted okra genotype with the highest number of seeds per fruit was NS7772 (G1) (61 seeds), followed by OH3312 (G3) (53 seeds), and green gold (47 seeds) had the lowest number of seeds per fruit (G2). G1 produced the most fruits per plant, followed by G3 and G2. G2 produced the fewest. The G1 treatment produced the largest fruit production per genotype, followed by the G3 treatment, while the G2 treatment produced the lowest fruit yield per plant among all (**Table 2**; **Supplementary Figures 4**, **5**). Mahapatra et al., 2007 and Simon et al., 2013 reported results that were comparable. The fruit production per plant is proportional to the number of branches, the number of fruits, and the weight of the fruit. Subsequently, based on the aforementioned findings, the grafted okra genotype NS7772 is optimal for okra growers in the Vellore region of Tamil Nadu in terms of attaining high-quality fruits with a larger yield and, consequently, bigger returns per unit area.

## Conclusions

In conclusion, our work revealed a potential role of grafting in mitigating drought stress in okra genotypes and the mechanisms by which grafted plants exhibit enhanced drought tolerance. The drought resistance mechanisms in grafted plants are related with the improvement in growth parameters, which results from a number of morphological modifications, physiological modifications, and gene expression changes in response to drought stress. In response to severe drought stress and to maintain a healthy state, the leaves of grafted okra plants revealed an increase in soluble sugar, proline, and protein content, antioxidant activity (APX, CAT, and SOD), and expression of their isozymes to scavenge reactive oxygen species (ROS). Nongrafted okra plants (NS7772, green gold, and OH3312) lack the ability to integrate ROS, soluble sugars, and proline in response to drought stress, hence displaying a drought-sensitive phenotype. However, by grafting them onto the drought-tolerant rootstock NS7774, the toxic effects of drought stress were mitigated resulting in drought-tolerant phenotype. In addition, the significance of grafting in the regulation of proteins involved in numerous biological functions and metabolic pathways may provide to a better understanding of the probable mechanism(s) plants may have evolved to mitigate the negative impacts of drought stress. Further analysis demonstrated that photosynthesis, metabolic activity, defence response, protein synthesis, and cell/cycle and division activities were the primary mechanisms governing the drought tolerance of grafted okra plants. Studies of the transcriptomes of drought-responsive genes *RD2*, *HAT22*, *PP2C*, *WRKY33*, *DREB1A*, and *DREAB1C* demonstrated an increase in gene expression in response to grafting under drought stress. This study suggests that comparative proteomics provides comprehensive insights into the mechanism through which okra rootstock confers drought tolerance. This work concludes by addressing the physiological and molecular mechanisms underlying the enhanced drought tolerance of grafted okra genotypes, hence enabling the large-scale production of okra in drought-prone regions.

## Data availability statement

The original contributions presented in the study are included in the article/**Supplementary Material**. Further inquiries can be directed to the corresponding author.

## Author contributions

SM conceptualized and supervised the work. KR performed the experiments and wrote the manuscript. SM edited and finalized the manuscript. All authors contributed to the article and approved the submitted version.
