# Supplementary material for: Grafting enhances drought tolerance by regulating and mobilizing proteome, transcriptome and molecular physiology in okra genotypes
Source: Front Plant Sci. 2023 May 12;14:1178935. doi: 10.3389/fpls.2023.1178935 (PMC10214962; doi:10.3389/fpls.2023.1178935)
Supplement: Supplementary file 1 [file Presentation_1.pdf]

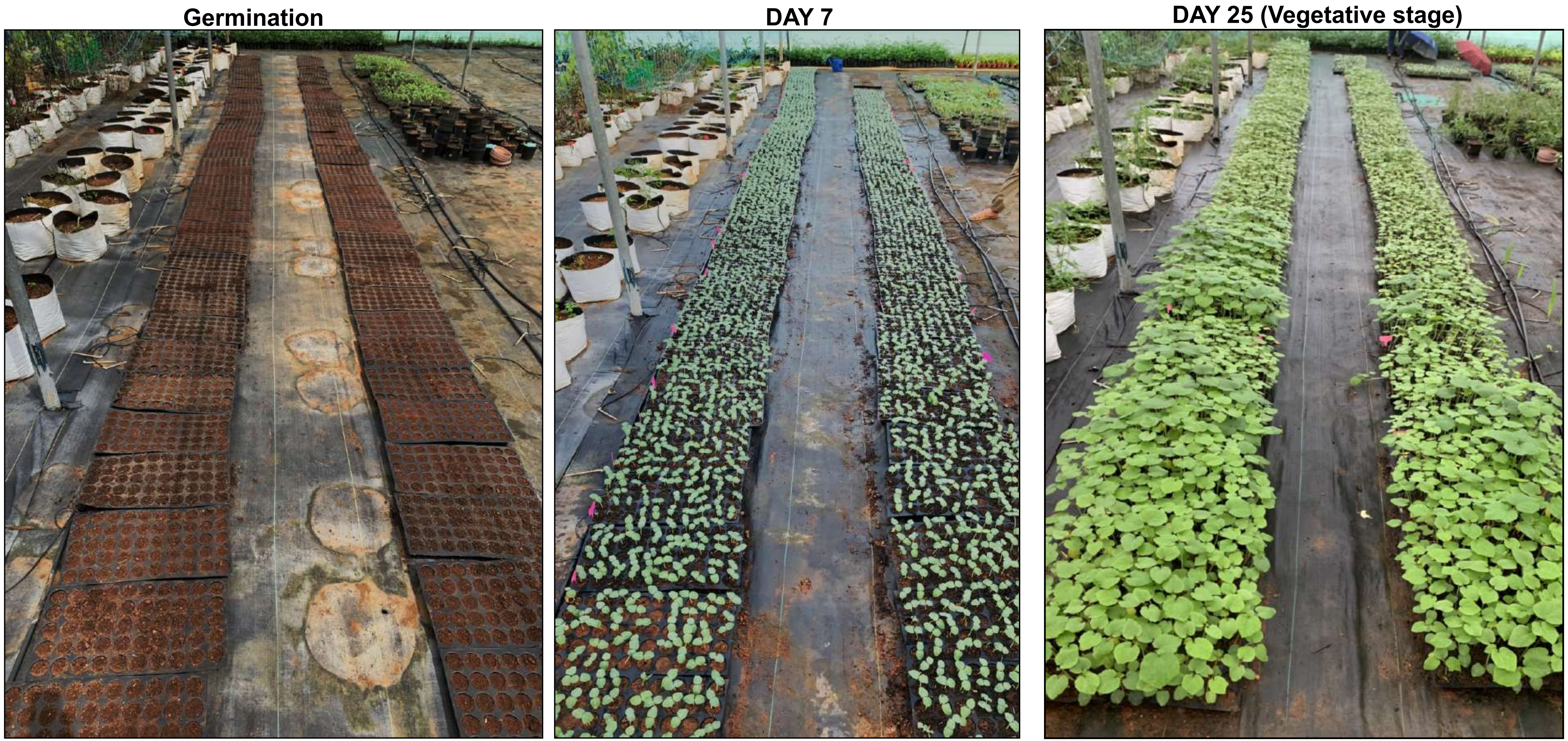

Figure 1 : Visual representation of various stages of okra genotypes for the yield study

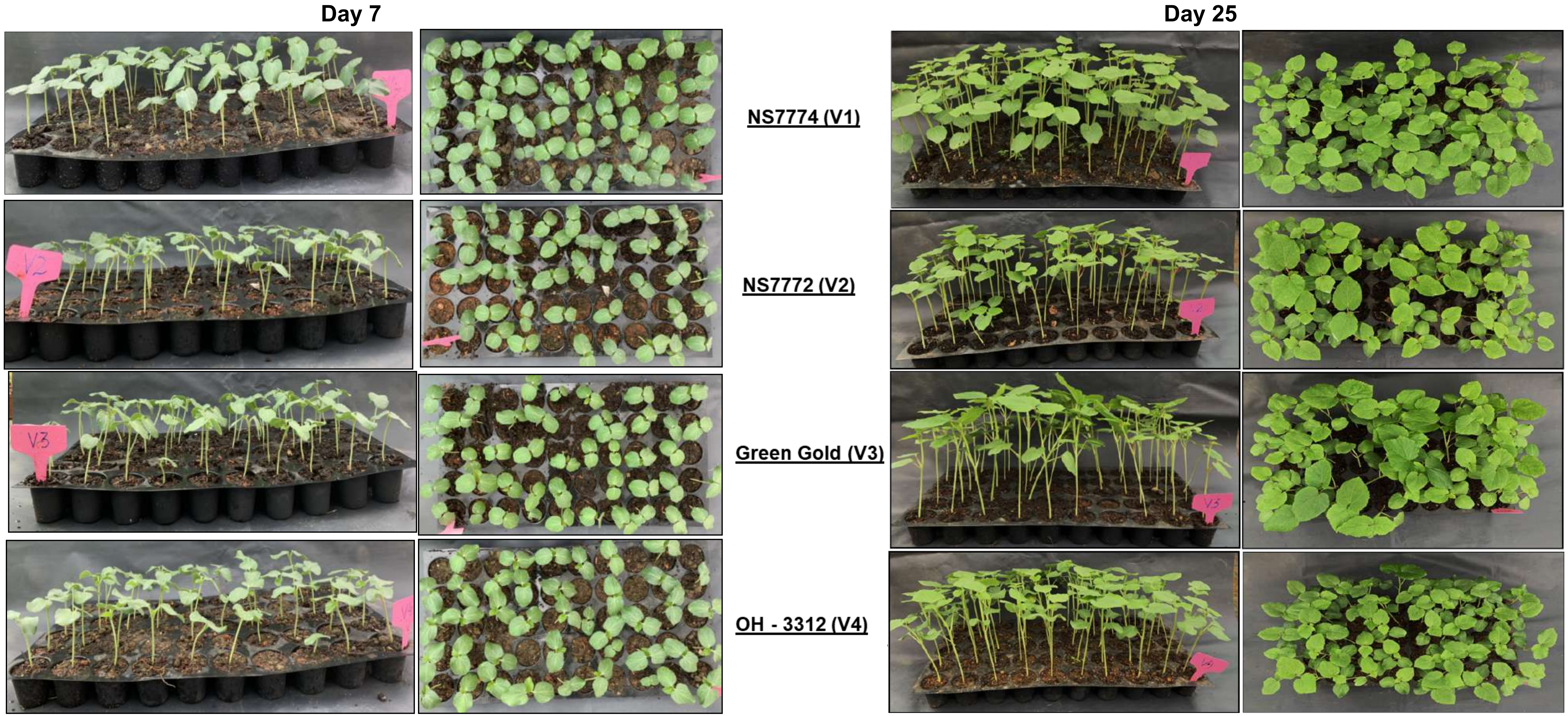

Figure 2 : Visual representation of early and later stage of vegetative stage of okra genotypes in portrays

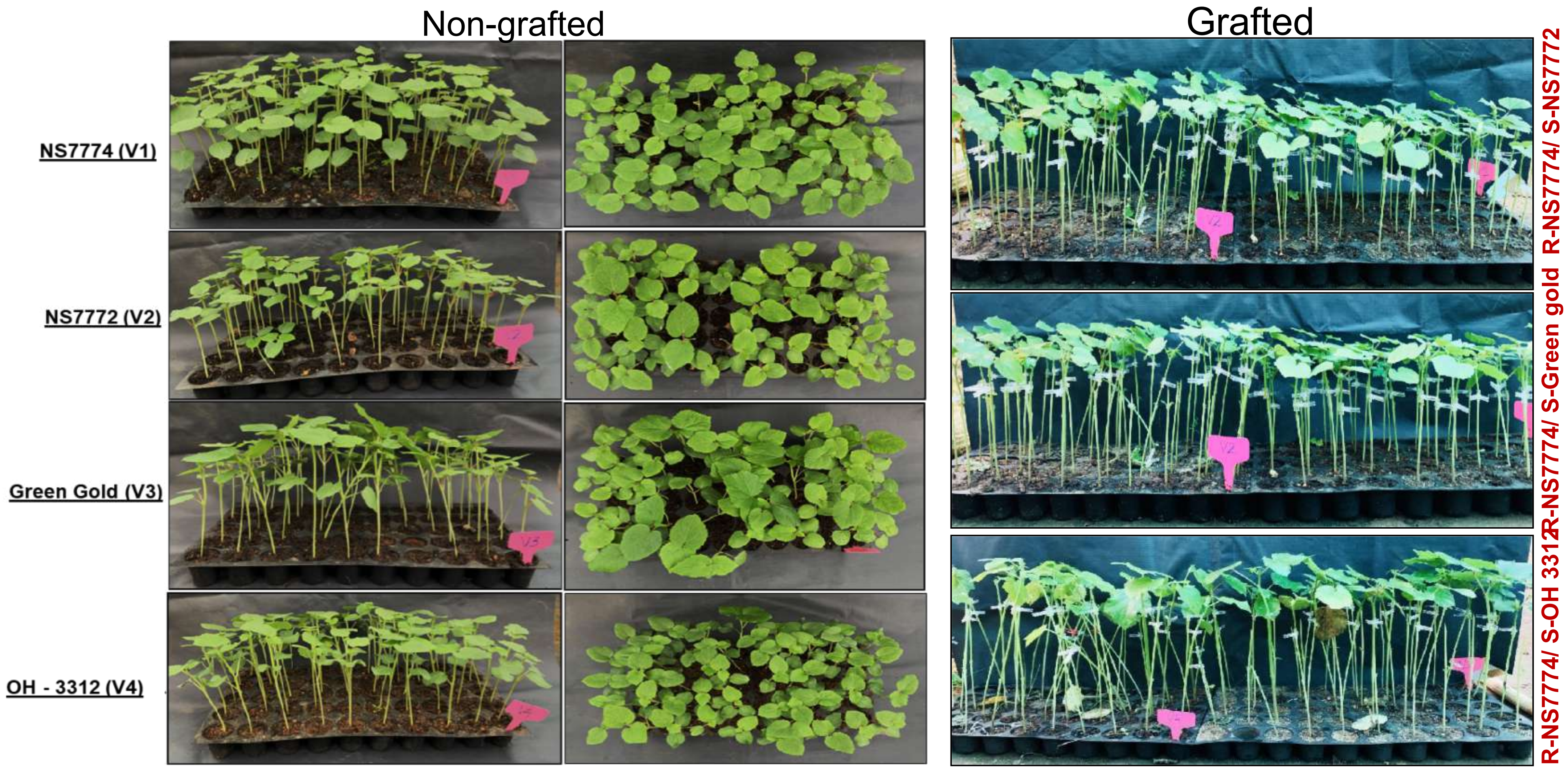

Figure 3 : Morphological representation of non-grafted okra genotypes (NS7774, NS7772, Green Gold and OH3312) and grafted okra genotypes (R-NS7774/S-NS7772, R-NS77774/S-Green Gold, R-NS7774/S-OH3312). Photographs represent the comparison among the non-grafted and grafted okra genotypes during their early vegetative phase.

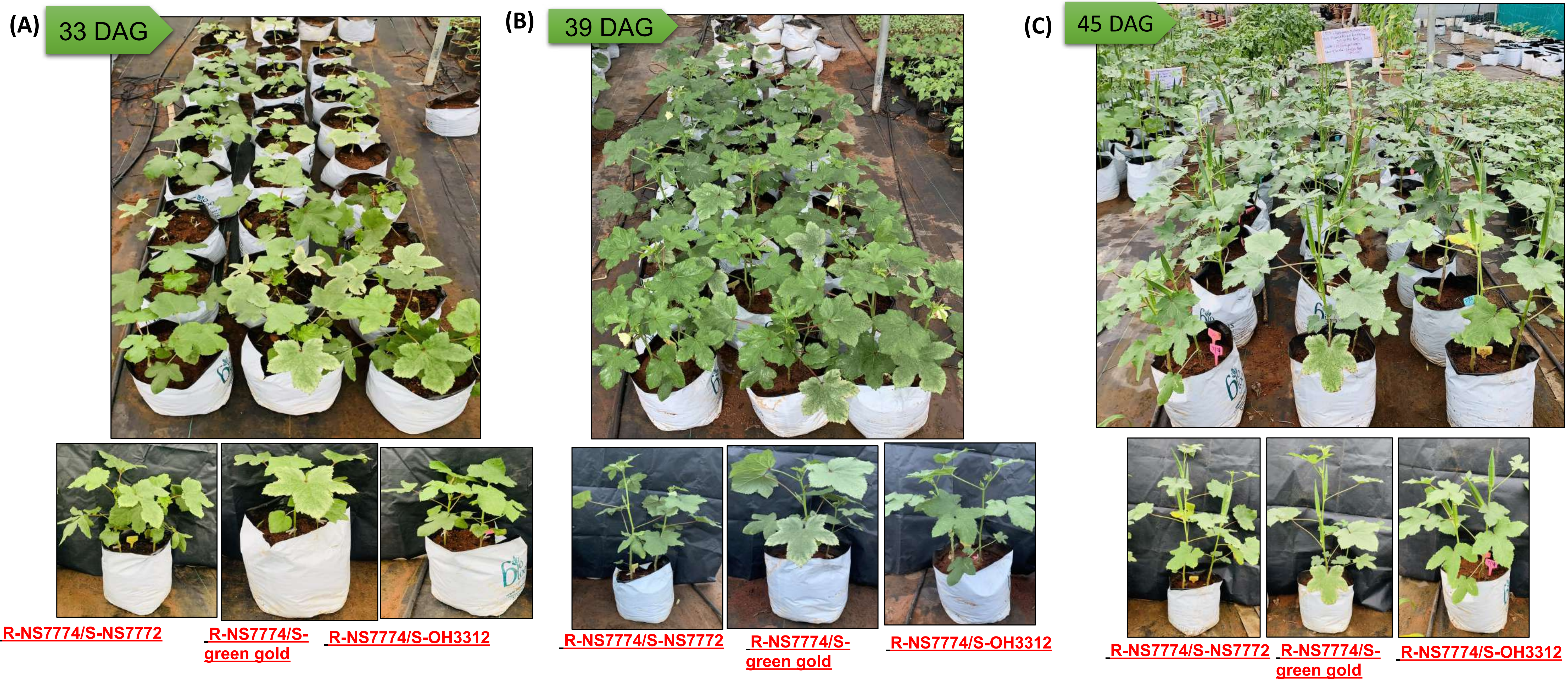

Figure 4 : Visual representation of grafted okra genotypes R-NS7774/S-NS7772, R-NS77774/S-Green Gold and R-NS7774/S-OH3312 at various stages of growth.

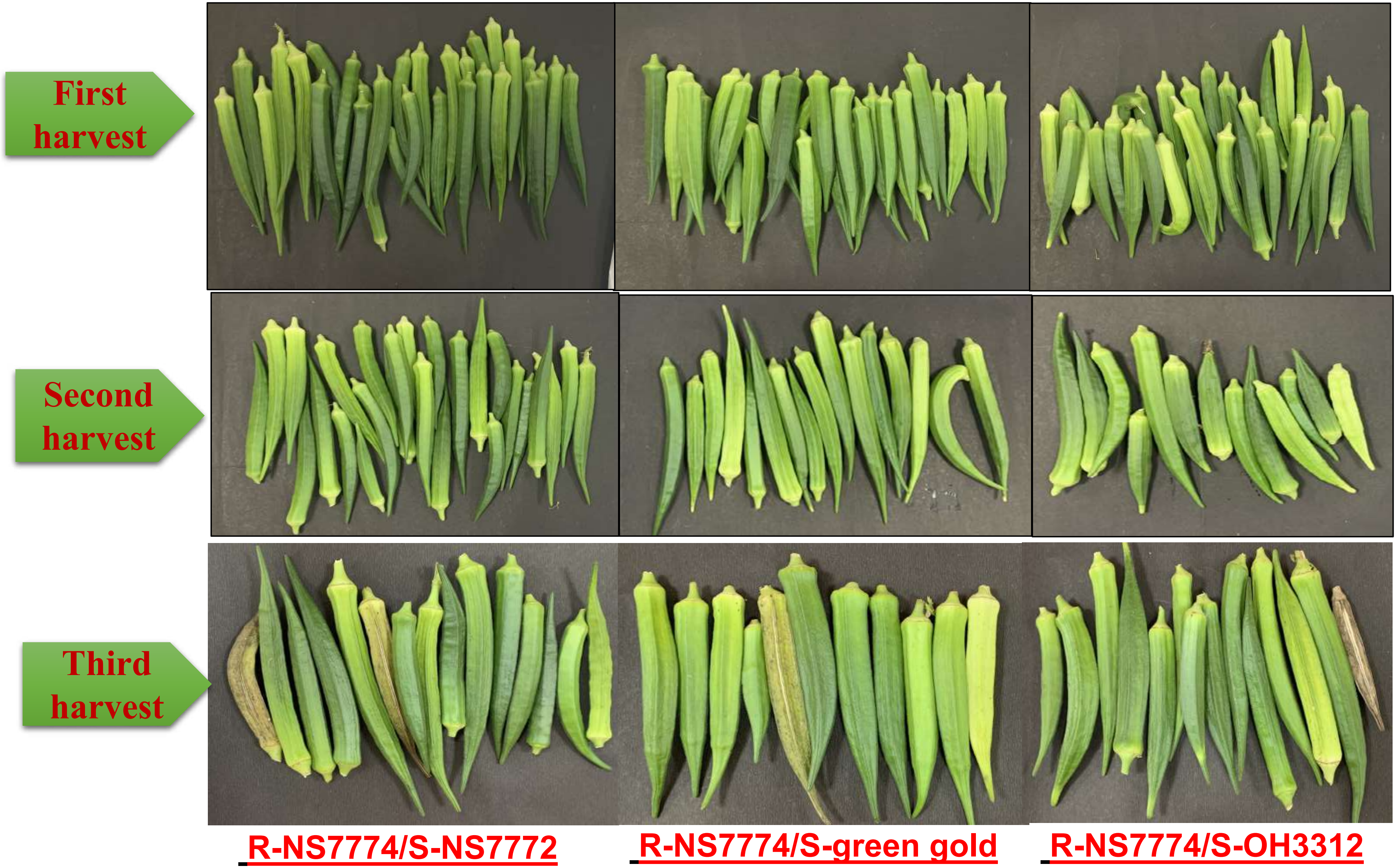

Figure 5 : Visual representation of the yield of grafted okra genotypes R-NS7774/S-NS7772, R-NS77774/S-Green Gold and R-NS7774/S-OH3312.
