# Supplementary material for: Grafting enhances drought tolerance by regulating and mobilizing proteome, transcriptome and molecular physiology in okra genotypes
Source: Front Plant Sci. 2023 May 12;14:1178935. doi: 10.3389/fpls.2023.1178935 (PMC10214962; doi:10.3389/fpls.2023.1178935)
Supplement: Supplementary file 2 [file Table_1.docx]

| **Gene** | **Forward sequence**  **(5’---------3’)** | **Reverse sequence**  **(5’---------3’)** |
| --- | --- | --- |
| *RD2* | GGGATGCTGGTAAGGTAA | TTATGACAGGTGCGGATT |
| *HAT22* | TTGAAGAGGTGTTGTGAGA | CAGTGATCTGAGTTCTTGTAA |
| *PP2C* | TGATTGTGATAACGCTGTTC | TTCTTCTACGGCTTCTGATT |
| *WRKY33* | TGATTTCAGCCAACACTTCA | ATCACTGGAGATGTCTTACA |
| *DREB1A* | AGTTATCCAAAGAGGCCCGC | CCATATCCGCCTTCGGGAAA |
| *DREB1C* | CCATGGAGAACCCATTTGTGC | TTGGAAGCAACCTCCTGCTG |
| *Actin* | ATCCTCCGTCTTGACCTTG | TGTCCGTCAGGCAACTCAT |

**Table T1:** List of Primers used in the study for RT-PCR

61
